# Crosstalk between ORMDL3, serine palmitoyltransferase, and 5-lipoxygenase in the sphingolipid and eicosanoid metabolic pathways

**DOI:** 10.1016/j.jlr.2021.100121

**Published:** 2021-09-22

**Authors:** Viktor Bugajev, Tomas Paulenda, Pavol Utekal, Michal Mrkacek, Ivana Halova, Ladislav Kuchar, Ondrej Kuda, Petra Vavrova, Björn Schuster, Sergio Fuentes-Liso, Lucie Potuckova, Daniel Smrz, Sara Cernohouzova, Lubica Draberova, Monika Bambouskova, Petr Draber

**Affiliations:** 1 Department of Signal Transduction, Institute of Molecular Genetics of the Czech Academy of Sciences, Prague, Czech Republic; 2 Research Unit for Rare Diseases, Department of Pediatrics and Adolescent Medicine, First Faculty of Medicine, Charles University and General University Hospital in Prague, Prague, Czech Republic; 3 Department of Metabolism of Bioactive Lipids, Institute of Physiology of the Czech Academy of Sciences, Prague, Czech Republic; 4 Czech Centre for Phenogenomics, Institute of Molecular Genetics of the Czech Academy of Sciences, Prague, Czech Republic; 5 CZ-OPENSCREEN, Institute of Molecular Genetics of the Czech Academy of Sciences, Prague, Czech Republic; 6 Department of Immunology, Second Faculty of Medicine, Charles University, Prague, Czech Republic

**Keywords:** sphingolipids, leukotrienes, immunology, signal transduction, inflammation, peritoneal-derived mast cells, ER membrane domains, lipid mass spectrometry, HPLC, ACN, acetonitrile, BMMC, bone marrow-derived mast cell, BMMCL, bone marrow-derived mast cell line, BSS, buffered saline solution, CCL, chemokine ligand, cDNA, complementary DNA, C1P, ceramide-1-phosphate, COX, cyclooxygenase, ER, endoplasmic reticulum, E/W, equilibration/washing, FcεRI, high-affinity IgE receptor, GSDMB, gasdermin B, GST, glutathione-*S*-transferase, HEK293FT, human embryonic kidney 293FT, HMC-1.1, human mast cell line 1.1, IL, interleukin, IκB-α, NF-κB inhibitor-α, 5-LO, 5-lipoxygenase, LT, leukotriene, LTC4S, LTC4 synthase, MEYS, The Ministry of Education, Youth and Sports, 2-ME, 2-mercaptoethanol, ORMDL3, ORM1-like protein 3, PDMC, peritoneal-derived mast cell, PGD2, prostaglandin D2, PLA2, phospholipase A2, SCF, stem cell factor, S1P, sphingosine-1-phosphate, SPT, serine palmitoyltransferase, STIM1, stromal interaction molecule 1, TNP, 2,4,6-trinitrophenol, TS, Twin-Strep tag

## Abstract

Leukotrienes (LTs) and sphingolipids are critical lipid mediators participating in numerous cellular signal transduction events and developing various disorders, such as bronchial hyperactivity leading to asthma. Enzymatic reactions initiating production of these lipid mediators involve 5-lipoxygenase (5-LO)-mediated conversion of arachidonic acid to LTs and serine palmitoyltransferase (SPT)-mediated de novo synthesis of sphingolipids. Previous studies have shown that endoplasmic reticulum membrane protein ORM1-like protein 3 (ORMDL3) inhibits the activity of SPT and subsequent sphingolipid synthesis. However, the role of ORMDL3 in the synthesis of LTs is not known. In this study, we used peritoneal-derived mast cells isolated from ORMDL3 KO or control mice and examined their calcium mobilization, degranulation, NF-κB inhibitor-α phosphorylation, and TNF-α production. We found that peritoneal-derived mast cells with ORMDL3 KO exhibited increased responsiveness to antigen. Detailed lipid analysis showed that compared with WT cells, ORMDL3-deficient cells exhibited not only enhanced production of sphingolipids but also of LT signaling mediators LTB_4_, 6t-LTB_4_, LTC_4_, LTB_5_, and 6t-LTB_5_. The crosstalk between ORMDL3 and 5-LO metabolic pathways was supported by the finding that endogenous ORMDL3 and 5-LO are localized in similar endoplasmic reticulum domains in human mast cells and that ORMDL3 physically interacts with 5-LO. Further experiments showed that 5-LO also interacts with the long-chain 1 and long-chain 2 subunits of SPT. In agreement with these findings, 5-LO knockdown increased ceramide levels, and silencing of SPTLC1 decreased arachidonic acid metabolism to LTs to levels observed upon 5-LO knockdown. These results demonstrate functional crosstalk between the LT and sphingolipid metabolic pathways, leading to the production of lipid signaling mediators.

Production of leukotrienes (LTs) and de novo sphingolipid biosynthesis are located in the cytosolic leaflet of the endoplasmic reticulum (ER) membranes and are catalyzed in the first step by rate-limiting enzymes 5-lipoxygenase (5-LO) and serine palmitoyltransferase (SPT), respectively (1, 2). The 5-LO-dependent signaling mediators are involved in bronchoconstriction (3), low-grade systemic inflammation accompanying type 1 diabetes (4), and impaired wound healing in type 1 diabetes (5). In contrast, the sphingolipid pathway, comprising sphingosine-1-phosphate (S1P) and ceramide-1-phosphate (C1P) as second messengers (6, 7), plays an important role in type 1 diabetes (8) and bronchial reactivity in the absence of inflammation (9).

The activity of SPT in mammals is regulated by the ORM1-like protein (ORMDL) family (10, 11, 12, 13) of highly conserved ER membrane proteins (ORMDL1-3) that are expressed in many tissues (14). In addition to its role in sphingolipid homeostasis (10, 11, 12, 13), ORMDL3 was found to be involved in Ca^2+^ homeostasis (15) and in facilitating unfolded protein response (15, 16). Previous genome-wide association studies showed that ORMDL3 is a risk factor of childhood-onset asthma and linked this disease with SNPs mapped in the 17q21 region (17, 18), where the human *ORMDL3* with other five coding genes resides (19). Other studies found that predisposition to primary biliary cirrhosis (20), type 1 diabetes (21), and Crohn's disease (22) are also associated with SNPs in the 17q21 region and decreased ORMDL3 mRNA levels in lymphoblastoid cell lines (23). In contrast, the risk alleles implicated in asthma (17, 18) are linked to increased levels of the *ORMDL3* gene transcript (17, 23).

*In vivo* experiments with mice showed that ORMDL3 deficiency prevented development of *Alternaria*-induced allergic airway response (24) and that enhanced expression of ORMDL3 resulted in increased levels of airway remodeling followed by an enhanced spontaneous response to methacholine (25). These findings were confronted by Debeuf *et al.* (26) using mice with enhanced or reduced ORMDL3 expression levels, exhibiting the expected changes in ceramides, but with no effects on experimental allergic asthma models. The complexity of ORMDL3 function in vivo was underlined by the study showing that selective deletion of ORMDL3 in the airway epithelium challenged with allergen unexpectedly exhibit increased airway hyperresponsiveness (27).

Experiments with mouse bone marrow-derived eosinophils showed that enhanced expression of ORMDL3 was accompanied by increased translocation of the phosphorylated p65 subunit of NF-κB into cell nuclei (28). Furthermore, lung epithelial cell line A549 transfected with ORMDL3 exhibited enhanced expression of chemokines C-X-C motif chemokine ligand 10, CXXL-11, interleukin (IL)-8, chemokine ligand 20 (CCL-20), metalloproteases a disintegrin and metalloproteinase domain-containing protein 8 and matrix metallopeptidase 9, and oligoadenylate synthetases (16). On the other hand, we have identified ORMDL3 as a negative regulator of the high-affinity IgE receptor (FcεRI) signaling (29). Antigen-activated bone marrow-derived mast cells (BMMCs) with ORMDL3 knockdown promoted phosphorylation of NF-κB inhibitor-α (IκB-α) followed by translocation of the p65 subunit of NF-κB into the nuclei and subsequent expression of proinflammatory cytokines, including TNF-α, IL-6, and IL-13, CC motif CCL3 and CCL4, and enhanced cyclooxygenase-2 (COX2)-dependent synthesis of prostaglandin D_2_ (PGD_2_) (29). Recently, we found that BMMCs isolated from mice deficient in both ORMDL2 and ORMDL3 exhibited increased sphingolipid levels and enhanced release of IL-4, IL-6, and TNF-α cytokines when compared with WT cells (30). Similarly, increased expression of IL-2 was observed in activated primary memory CD4^+^ T cells with silenced ORMDL3 (31). These and other findings of frequently opposite roles of ORMDL3 in various cell types in vitro (19, 28, 29, 31) and in vivo (24, 25, 26, 27) suggested that ORMDL3 could be involved in other signal transduction pathways than previously thought.

In this study, we examined the role of ORMDL3 in LT and sphingolipid metabolic pathways using peritoneal-derived mast cells (PDMCs) from WT and ORMDL3 KO mice. We found that PDMCs deficient in ORMDL3 exhibited increased activation of the FcεRI/IκB-α/TNF-α signaling axis and that endogenous ORMDL3 limited not only the SPT-dependent pathway but also the synthesis of LTs LTB_4_, 6t-LTB_4_, LTC_4_, LTB_5_, and 6t-LTB_5_. To investigate the new ORMDL3-dependent pathways, we immunoprecipitated tagged ORMDL3 from mast cells and found 5-LO as an interacting partner. Moreover, we found that 5-LO forms complexes with SPTLC1, SPTLC2, and ORMDL3. Changes in the composition of the complexes resulted in altered production of both, LTs and sphingolipids. The combined data indicate extensive crosstalk between the metabolic pathways directed by 5-LO and SPT.

## Materials and methods

### Reagents

All reagents were purchased from Sigma-Aldrich/Merck if not stated otherwise.

### Antibodies

Antibodies specific for hypoxanthine phosphoribosyltransferase (sc-376938), actin (sc-8432), IκB-α (sc-371), and m-α-SPTLC1 (sc-374143) were obtained from Santa Cruz Biotechnology. Antibodies against SPTLC1 (ab176706) and SPTLC2 (ab23696) were obtained from Abcam. Antibodies against 5-LO (catalog no. #3289), phospho-IκB-α (catalog no. #9246), and MYC tag (catalog no. #2276) were purchased from Cell Signaling Technology. Antibodies against HA tag (NB600-363) and stromal interaction molecule 1 (STIM1) (NB110-60547S) were obtained from Novus Biologicals. Antibody against FLAG tag (F1804) was obtained from Sigma-Aldrich. Antibodies against 5-LO (catalog no. #610695; used for microscopy analysis) and mouse-specific TNF-α-conjugated to R-phycoerythrin (catalog no. #561063) were from Becton Dickinson. Antibodies against FcεRI-conjugated to FITC (#11-5898) and c-Kit (CD117) conjugated to allophycocyanin (catalog no. #17-1171) were from eBioscience. HRP-conjugated goat anti-mouse IgGs were from Santa Cruz Biotechnology (sc-2005) or Jackson ImmunoResearch Laboratory (catalog no. #115-035-003), and HRP-conjugated goat anti-rabbit IgGs were from Santa Cruz Biotechnology (sc-2004) or Jackson ImmunoResearch Laboratory (catalog no. #111-035-144). SPTLC2-specific polyclonal antibody (ab23696) presumably recognizes a nonspecific band not associated with membrane fractions, which is approximately 5 kDa higher than the membrane-bound SPTLC2 subunit (supplemental Fig. S4B). ORMDL3 Twin-Strep tag (TS) or TS-5-LO also interacts exclusively with the lower band corresponding to SPTLC2. Specific pan-ORMDL polyclonal serum was obtained from immunized rabbit as previously described (29). These antibodies recognize all members of the ORMDL family at least of murine, human, and rat origin. IgE monoclonal antibody specific for 2,4,6-trinitrophenol (TNP), clone IGEL b4 1, was produced in our laboratory from hybridoma cells obtained from Dr A. K. Rudolph (32).

### Cells

PDMCs were isolated after washing the peritoneum of ORMDL3 KO and WT mice with 15 ml of culture medium. The cells were cultured in RPMI-1640 medium (R8758; Sigma-Aldrich) supplemented with antibiotics (100 U/ml penicillin, 100 μg/ml streptomycin; Gibco), 71 μM 2-mercaptoethanol (2-ME), MEM nonessential amino acids, 0.7 mM sodium pyruvate, 2.5 mM l-glutamine, 12 mM d-glucose, 10% fetal bovine serum (Biosera; catalog no. FB-1090/500), and 4% WEHI-3 culture supernatant as a source of IL-3 and 4% culture supernatant from Chinese hamster ovary cells transfected with the complementary DNA (cDNA) encoding murine stem cell factor (SCF), a kind gift of Dr P. Dubreuil; INSERM, Marseille, France). PDMCs were grown for 2 weeks, tested for the presence of c-Kit (CD117) and FcεRI on the cell surface, and then used within 2 months for the experiments. A stable cell line derived from bone marrow-derived mast cell line (BMMCL) of C57BL/6 origin was donated by Dr M. Hibbs (Ludwig Institute for Cancer Research, Melbourne, Australia) and characterized in previously published studies (33, 34). The cells were cultured in RPMI-1640 supplemented as aforementioned except that SCF was omitted. The human mast cell line 1.1 (HMC-1.1) was obtained from the Mayo Foundation for Medical Education and Research (MAYO). The cells were cultured in Iscove's modified Dulbecco's medium supplemented with fetal bovine serum (10%), l-glutamine (2 mM), penicillin (100 U/ml), and streptomycin (100 mg/ml). The human embryonic kidney 293FT (HEK293FT) cell line was provided by the laboratory of Dr J. Rivera (National Institutes of Health, Bethesda, MD) and cultivated in DMEM (D6429; Sigma-Aldrich) supplemented with antibiotics (100 U/ml penicillin, 100 μg/ml streptomycin), 10% FCS (Biosera; catalog no. FB-1090/500), and 2.5 mM l-glutamine.

### Animal models

All mice were maintained and used in accordance with the Institute of Molecular Genetics guidelines (permit number 12135/2010-17210) and national guidelines (2048/2004-1020). We described the generation of mice deficient in ORMDL3, screening of founder animals, and the strategy of genotyping elsewhere (30).

### Cell activation

Cells were washed and activated in buffered saline solution (BSS; 135 mM NaCl, 5 mM KCl, 1.8 mM CaCl_2_, 5.6 mM glucose, 20 mM Hepes, pH 7.4) supplemented with 0.1% essentially fatty acid-free and globulin-free albumin from bovine serum (BSA; Sigma; A7030). Mast cells were sensitized with TNP-specific mouse IgE, clone IGEL b4 1 at final concentration 1 μg/ml (32). IgE bound to FcεRI was crosslinked with antigen (TNP-BSA conjugate, 15–25 mol TNP/mol BSA; prepared in our laboratory). Ionomycin (Thermo Fisher Scientific; I24222) was used to induce activation in colocalization study. Thapsigargin (Thermo Fisher Scientific; T7459), an inhibitor of the SERCA pump, was used to induce the Ca^2+^-dependent cell response, independent of FcεRI triggering.

### Immunoblotting

Whole-cell extracts were prepared by washing the cells in cold PBS, pH 7.2, followed by solubilizing them in hot SDS-PAGE sample buffer (35), sonicating three times for 5 s at 20^o^C, and boiling for 5 min. Alternatively, in experiments analyzing sphingolipids or studies aimed to quantify expression of selected proteins (load), 2× concentrated SDS-PAGE sample buffer was added to the aliquots of sonicated lysates. Protein extracts (25 μg) were size fractionated in 13% SDS-PAGE gels and electrophoretically transferred onto nitrocellulose membrane (0.45 μm; GE Healthcare Life Sciences) using a Bio-Rad Mini-PROTEAN apparatus. The membranes were blocked with 2% BSA-TNT solution (20 mM Tris-HCl, pH 7.5, 200 mM NaCl, and 0.1% Tween) and analyzed by immunoblotting with protein-specific antibodies. Bound primary antibodies were detected with the corresponding HRP-conjugated secondary antibodies. HRP signal in loads and phosphorylation studies was detected with chemiluminescence reagent (36), and HRP signals in pull-down studies were developed with WesternBright ECL HRP substrate (Advansta) or SuperSignal™ West Femto (Thermo Fisher Scientific) and collected with Luminescent Image Analyzer LAS 3000 (FujiFilm). Aida Image Analyzer, version 5.0 software (Raytest) was used for signal quantification.

### Generation of stable knockdowns or overexpressors using lentiviral transduction

Lentiviruses suitable for transduction were prepared by mixing 1.5 ml aliquots of Opti-MEM medium (Thermo Fisher Scientific) with 21 μl of ViraPower Lentiviral Packaging Mix (Thermo Fisher Scientific), 14 μg of lentiviral construct, and 105 μl of polyethylenimine (1 mg/ml; 25 kDa; linear form; Polysciences). The mixture was incubated for 20 min at room temperature before it was added to HEK293FT packaging cells in 22 ml of DMEM culture medium into a 175 cm^2^ flask. Seventy-two hours later, the virus-containing medium was centrifuged at 813 *g* at room temperature, followed by filtering through 0.22 μm pore size nitrocellulose filters (GE Healthcare). BMMCL cells were washed in the culture medium before transduction and then suspended in the medium supplemented with protamine (1 μg/ml; Sigma-Aldrich) at a concentration of 2 × 10^6^/ml. One day before transduction, HEK293FT cells were seeded onto a 24-well plate at 50% confluence. Just before transduction, the culture medium was exchanged by medium with protamine (1 ml). Both cell types were transduced with 0.5 ml of supernatant aliquots containing lentiviral particles. After 24 h, HEK293FT cells were washed with the medium supplemented with 5 μg/ml of puromycin (Apollo Scientific). Forty-eight hours after transduction, HEK293FT and BMMCL cells were passaged in the presence of 5 and 3 μg/ml of puromycin, respectively. One day before the experiment, the cells were washed in medium without puromycin. In all experiments, more than 90% of cells were alive, and the selected populations were maintained for less than 6 weeks in culture.

### Immunoprecipitation and protein identification by mass spectrometry analysis

The cells (1 × 10^9^) were activated in 6 ml of BSS-BSA with 1 μM thapsigargin for 3 min and then lysed in 4 ml of 1% TRITON-X100 in Tris-HCl buffer (20 mM Tris, 5 mM NaCl, 2 mM EDTA, and pH 7.4) supplemented with 1 mM Na_3_VO_3_, 1 mM PMSF, and protease inhibitor cocktail (1:100; P8340; Sigma-Aldrich) at 4°C for 30 min. For precipitation, protein G-coated beads armed with anti-MYC antibody (20 μl; catalog no. #2276) were used. Lysates were incubated with the beads for 2 h at 4°C, and the precipitates were eluted with 1× SDS-PAGE loading buffer containing 2-ME. Eluates were size fractionated in 10% SDS-PAGE gel. Coomasie-stained gels were analyzed, and bands specifically enriched only in the precipitates from ORMDL3-MYC cell lysates, when compared with cells expressing empty pCDH vector, were excised from the gels. Fragments were cut into small pieces and decolorized in sonic bath at 60°C several times with 0.1 M 4-ethylmorpholine acetate (pH 8.1) in 50% acetonitrile (ACN). After complete destaining, proteins were reduced by 50 mM Tris(2-carboxyethyl) phosphine in 0.1 M 4-ethylmorpholine acetate (pH 8.1) for 5 min at 80°C and alkylated using 50 mM iodoacetamide in 0.1 M 4-ethylmorpholine acetate (pH 8.1) for 30 min in the dark at 22°C. Then, the gel was washed with water, ACN, and then partly dried using a SpeedVac concentrator (Savant). Finally, the gel was reconstituted with cleavage buffer containing 0.01% 2-ME, 0.05 M 4-ethylmorpholine acetate (pH 8.1), 10% ACN, and sequencing-grade trypsin (Promega; 10 ng/μl). Digestion was carried out overnight at 37°C, and the resulting peptides were extracted with 30% ACN/0.1% trifluoroacetic acid and subjected to mass spectrometric analysis. Mass spectra were acquired in the positive ion mode using MALDI-FTMS APEX Qe (Bruker Daltonics) equipped with 9.4 T superconducting magnet and SmartBeam laser. The acquisition mass range, *m/z*, was 250–4,000, and 2 M data points were collected. The instrument was externally calibrated using PepMix II peptide standard (Bruker Daltonics). Typical mass accuracy was below 3 ppm. A saturated solution of α-cyano-4-hydroxy-cinnamic acid in 50% ACN/0.2% trifluoroacetic acid was used as a MALDI matrix. One microliter of the matrix solution was mixed with 1 μl of the sample on the target, and the droplet was allowed to dry at ambient temperature. After analysis, the spectra were apodized using sin apodization with one zero fill. The spectra deisotoping and peak list was generated using DataAnalysis, version 5.0 SR1 (Build 203.2.3586; Bruker Daltonics). Proteins were identified by peptide mass fingerprinting using the search algorithm MASCOT (version 2.7; Matrix Science) and/or mMass (version 5.5.0) (37). The mass error tolerance was set to 5 ppm. Carbamidomethylation of cysteine and oxidation of methionine were selected as fixed and variable modification, respectively. The number of miscleavages was set up to 2 for trypsin considering the specificity on the C termini of any arginine and lysine. Mass data were searched against the mammals SwissProt database (UniProtKB: 2021_03).

### Strep-Tactin affinity purification

Control and antigen (TNP-BSA)-activated BMMCL (7.5 × 10^7^/sample) and thapsigargin-activated HEK293FT cells (80–90% confluent, grown on 150 mm Petri dish/two samples) transduced with TS-tagged proteins alone and with FLAG-ORMDL3 or HA-ORMDL3, respectively, were lysed in 1.8 ml of ice-cold lysis buffer (50 mM Hepes-KOH, 150 mM KOAc, 2 mM MgOAc, 1 mM CaCl_2_, and pH 6.8) supplemented with 1 mM Na_3_VO_3_, 1 mM PMSF, 10 mM 1,10 phenanthroline, protease inhibitor cocktail (1:100; P8340; Sigma-Aldrich), glutathione 150 μM (BMMCL only), and 1% CHAPS (Apollo Scientific). Cells in the lysis buffer were efficiently disrupted by passing through an insulin syringe three times. Lysates were then rotated for 20 min at 4°C, then centrifuged at 16,100 *g*. Postnuclear supernatants were transferred into fresh tubes, centrifuged twice at 16,100 *g*, 4°C for 5 min. Aliquots (100 μl) were transferred to a tube containing 100 μl of 2× SDS-PAGE loading buffer with +2ME (used for immunoblots to detect loads; the input of HEK293FT cells corresponded to 3–5% of the whole material present in cell lysates). To the lysates, 50 μl of washed Strep-Tactin Superflow 50% resin (IBA) in lysis buffer without supplements was added (25 μl bed volume). After 20 min of incubation by slow rotation at 4°C, the resin was washed in lysis buffer with 0.25% CHAPS and 150 μM glutathione (BMMCL only) in three repeated steps. For elution of bound material, we used +2ME containing SDS-PAGE sample buffer. Samples were heated at 95°C for 10 min, size fractionated in 13.5% SDS-PAGE, and analyzed by direct immunoblotting.

### Production and isolation of glutathione-*S*-transferase-tagged proteins in bacteria

C41 and C43 bacteria used for protein production were obtained from Lucigen. C41 bacteria were used for glutathione-*S*-transferase (GST)-5-LO expression. C43 bacteria were transfected with expression vectors GST-ORMDL3, GST-LTC_4_ synthase (LTC4S), GST-FLAP, or empty pGEX-4T-2 vector to express GST alone. All bacteria strains were cultured in Luria-Bertani broth in the presence of ampicillin (100 μg/ml) at 37°C until the absorbance at 600 nm reached 0.8–1.0. Cultures were then transferred to 12°C. Protein expression was induced by adding isopropyl β-d-1-thiogalactopyranoside (final concentration of 1 mM) for 6 h. Bacteria were collected by centrifugation in Avanti J25I (Beckman Coulter) using JLA-10, 500 rotor at 8,000 rpm for 15 min at 4°C. Pellets were resuspended in an equilibration/washing (E/W) buffer containing 125 mM Tris-HCl, pH 8.0, 150 mM NaCl, and supplemented with 1% CHAPS, 1% Nonidet P-40, 1 mM PMSF, and a protein inhibitory cocktail from Promega. Bacteria were disrupted by 40 sonication cycles, each lasting 15 s, at amplitude 7, followed by 15 s pause on ice. Sonicates were centrifuged in a table centrifuge at 4°C, 13,200 *g* to remove the debris. Lysates were aliquoted and frozen in liquid nitrogen until further use.

### In vitro translation

^35^S-labeled proteins were prepared using the TNT® Quick coupled Transcription/Translation system (Promega) according to the manufacturer's protocol. In brief, TNT® T7 Quick Master Mix was mixed with plasmid template DNA 1 μg/50 μl reaction and ^35^S-labeled l-methionine (0.4 mCi/ml; Isotop). Samples were incubated for 1 h at 30°C. ^35^S-labeled proteins were directly used in the binding assays.

### GST pull-down and protein visualization

Glutathione agarose beads (Thermo Fisher Scientific; 50 μl per sample; 25 μl bed volume) were washed twice with E/W buffer. The beads were resuspended in 1 ml of E/W buffer, and bacterial lysate containing GST-tagged protein was added. Samples were incubated for 1 h at 4°C while rotating. Then, the beads were collected by short spin centrifugation (2,200 *g*, for 10 s). The supernatant was discarded, and the pellet was resuspended in E/W buffer. The washing step was repeated twice. Beads were then resuspended in Hepes-KOH buffer (50 mM Hepes-KOH, 150 mM KOAc, 2 mM Mg(OAc)_2_·4H_2_O, 1 mM CaCl_2_, and pH 6.8) supplemented with 1% CHAPS and 5 mM DTT. In vitro-translated ^35^S-methionine-labeled protein was added to the agarose beads with bound GST-tagged proteins. Samples were incubated for 1 h at 4°C while rotating at the minimum speed. After that, the beads were collected by short spin (2,200 *g*, for 10 s) and washed three times with Hepes-KOH buffer supplemented with 0.1% CHAPS and 5 mM DTT. Bound proteins were recovered from the beads by boiling in SDS-PAGE sample buffer. Protein samples were then size fractionated in 13% SDS-PAGE. To visualize the proteins, the gels were stained with Coomassie brilliant blue. Subsequently, the gels were soaked in ethanol (50% v/v) supplemented with glycerol (5% v/v) at 20°C for 1 h, followed by drying using a gel drier (SCIE-PLAS) at 80°C for 1.5 h. ^35^S-labeled proteins were then visualized by exposing on the BAS-MS 2025 imaging plate (FujiFilm) for 72 h and scanned in an FX scanner (Bio-Rad) using software Quantity one (Bio-Rad).

### Subcellular fractionation

The procedure was done as previously described (38) with some modifications. BMMCL cells (1 × 10^7^) were washed twice with BSS-BSA and activated with 1 μM thapsigargin at 37^o^C. Cells were then spun down and resuspended in 1 ml of ice-cold TKM buffer (50 mM Tris-HCl, pH 7.4, 25 mM KCl, and 5 mM MgCl_2_) with protease inhibitors (1 mM PMSF and protease inhibitor cocktail from Sigma-Aldrich). Cells were lysed by nitrogen cavitation at 350–400 psi applied for 5 min using a cell disruption chamber (Parr Instrument Company; Model 4639; cell disruption vessel). The cell lysate was centrifuged at 1,000 *g*, 4^o^C, to separate nuclei and a postnuclear supernatant. Postnuclear supernatants were collected and centrifuged in ultracentrifuge OptimaMax, rotor MLA-130, 100,000 *g* for 1 h to separate the cytosolic (C) and membrane (M) fraction. Membrane fractions were washed with 1 ml of ice-cold TKM buffer to avoid potential contamination by the cytosolic fraction. In the meantime, nuclear pellets were resuspended in 1 ml of TKM supplemented with 0.25 M sucrose, followed by sonication and ultracentrifugation as aforementioned to obtain the nuclear soluble (Ns) and nuclear membrane/pellet fraction (Np). All pellets were then sonicated in 1 ml of 1× SDS-PAGE sample buffer with 2-ME. To all supernatants, 250 μl of 4× SDS-PAGE sample buffer with 2-ME was added, and then they were sonicated. Before loading into wells of the gel, samples were denatured at 98^o^C for 5 min.

### Measurement of eicosanoids

Mast cells (4 × 10^6^) were sensitized with TNP-specific IgE (1 μg/ml) in SCF- and IL-3-free culture medium for 16 h. Then, the cells were washed in BSS-BSA and activated 30 min with antigen (TNP-BSA; 1 μg/ml) in 500 μl. To assess the production of PGD_2_, PDMCs were activated for 4 h. Cell supernatants were collected and immediately frozen in liquid nitrogen. The eicosanoids were measured only in antigen-activated cells, as in nonactivated cells, the levels of eicosanoids were under the limit of detection (data not shown). Pellets were used for assessment of protein expression by immunoblotting. The effect of fumonisin B_1_ (Santa Cruz Biotechnology; sc-201395) was produced according to Balsinde *et al.* (39) with some modifications. Briefly, PDMCs were pretreated before antigen activation with 1 μM fumonisin B_1_ for 45 min or 10 μM myriocin (Merck; M1177) overnight. Sample preparation and analysis of oxylipins were performed according to a published method (40). Supernatants of activated cells (100 μl) were thawed on ice, and aliquots of 450 μl of ice-cold methanol with 0.1% butylated hydroxytoluene and 0.1% acetic acid were added. Samples were sonicated for 10 s in sonicating baths, spiked with deuterated internal standards (5 ng total; Cayman Chemicals), and stored at −80°C for 1 h to promote protein precipitation. Precipitated material was removed by centrifugation (12,000 *g*, 10 min, 4°C), and the supernatant was mixed with water to get a final concentration of 10% methanol. Lipid metabolites were extracted using Strata-X 33u reverse phase extraction columns (Phenomenex; 100 mg/3 ml) according to the manufacturer's directions. Briefly, columns were washed with 3 ml of ethyl acetate, followed by 3 ml of 100% methanol and 3.5 ml of 10% methanol. Samples were loaded to columns and washed with 6 ml of 10% methanol followed by 3 ml of hexane. Eicosanoids were eluted with 2 ml of ethyl acetate and 2 ml of methanol, dried under a fine stream of nitrogen, resuspended in 200 μl solvent A_EICO (see later), and stored at −80°C or measured immediately.

An aliquot of 10 μl sample was injected in the UPLC system (UltiMate 3000 Binary RSLC System; Thermo Fisher Scientific) equipped with a Kinetex reverse-phase C18 column (150 × 2.1 mm, 100A, 1.7 μm; Phenomenex). Analytes were separated using gradient elution with stable flow of 300 μl/min at 50°C. Solvent A_EICO consisted of water-ACN-acetic acid (70:30:0.02; v/v/v), whereas solvent B_EICO consisted of ACN-isopropyl alcohol (50:50, v/v). The gradient program used to separate the eicosanoids was as follows: 0.5 min (0% solvent B_EICO), 1.73 min (23% solvent B_EICO), 6.53 min (45% solvent B_EICO), 7.73 min (60% solvent B_EICO), 10.73 min (75% solvent B_EICO), 11.03 min (90% solvent B_EICO), 11.93 min (90% solvent B_EICO), 12.50 min (100% solvent B_EICO), 13.00 min (0% solvent B_EICO), and 16.00 min (0% solvent B_EICO). A linear gradient was maintained between each step. All chemicals were of mass spectrometry grade.

An AB-Sciex (Foster City, CA) 5500 QTRAP hybrid, triple quadrupole, linear ion trap mass spectrometer equipped with a Turbo V ion source was used for all mass spectrometry analyses. The Analyst 1.6.1 software package was used to operate the mass spectrometer. Nitrogen gas was used as the collision gas for all metabolites. Eicosanoids were detected in negative electrospray ion mode—reaction monitoring—with the following source parameters: CUR = 25 psi, GS1 = 40 psi, GS2 = 50 psi, IS = −4500 V, CAD = HIGH, TEMP = 550°C, ihe = ON, EP = −10 V, and CXP = −15 V. For multiple reaction monitoring transitions, see Kuda *et al.* (40).

Standard curves were generated for oxylipins using mixed primary standard stocks (Cayman Chemicals) at seven different concentrations (0.005–500 ng/ml) and determined by generating a linear regression trend line.

### Measurement of sphingolipids

Mast cells (6 × 10^6^) were collected, washed in BSS-BSA buffer, and then in distilled water of chromatography grade (Sigma-Aldrich). Each cell pellet was suspended in 400 μl of distilled water and sonicated three times for 5 s. One part of the sonicate was saved to detect proteins by immunoblotting. Concentrations of the proteins were determined by the Bradford assay (Thermo Fisher Scientific). Samples containing 40 μg of proteins were placed into glass vials containing standards (C17:1 sphingosine [catalog no.: 860640P] and C17:0, d18:1 ceramide [catalog no.: 860517P] Avanti Polar Lipids). Then, 1 ml of 2:1 chloroform/methanol solution was added. Samples were shaken horizontally for 1 h at 22°C followed by filtration through Millex LH 0.45 μm filters (Merck Millipore).

Normal-phase HPLC was performed on a Silica-C column 5 cm × 2.1 mm, 4 μm 100A (catalog no.: 40000-05-P-2; MicroSolv Technology Corporation). Isocratic elution was done in a mobile phase consisting of methanol (BioSolve) with 5 mM of ammonium acetate. The elution program for isocratic HPLC separation was as follows: flow rate of 100 μl/min for 5 min.

Mass spectra were measured in an AB/MDS SCIEX API 4000 tandem mass spectrometer (SCIEX: AB Sciex LLC) equipped with an ESI source and coupled to an Agilent HPLC 1290 series (Agilent Technologies). Samples (20 μl in methanol with 5 mM ammonium acetate) were introduced by autosampler. The Analyst 1.6.3 software (AB Sciex, LLC) was used to operate the instruments and process the data. Ceramides and sphingosines were analyzed by selected reaction monitoring in the positive ion mode via generating the [M + H^+^]^+^ precursor ions. The product ion used for ceramide was *m/z* 264.4 corresponding to C18:1 sphingoid base, whereas the sphingosine product ion was [M − H_2_O + H^+^]^+^. Delays between mass transitions were set to 5 ms with Q1 and Q3 operating at unit mass resolution. The settling time was set to 500 ms. The curtain gas pressure was 20 psi. Nitrogen was used as the collision gas with pressure set at 10 psi. The capillary spray voltage was 5.5 kV. The temperature of the nebulizing gas (nitrogen) was 200°C. The ion source gas pressure was adjusted to 20 psi for source gas 1 and 50 psi for source gas 2. The interface heater was turned on during the analysis. The ion optics settings for ceramide molecular species measurements were 50 V for the declustering potential and 10 V for the entrance potential. The collision energy was set to 36 V with a collision cell exit potential of 5 V. Dwell time for each ion transition was set to 100 ms. For sphingoid base molecular species, the ion optics setting was as follows: declustering potential was set to 40 V and entrance potential was set to 10 V. The collision energy was set to 17 V with a collision cell exit potential of 5.5 V. Dwell time for each ion transition was set to 300 ms.

Concentrations were calculated using a known amount (12.5 ng) of internal standards C17:0, d18:1 ceramide (molecular weight = 551.6), and C17:1 sphingosine (molecular weight = 285.4) added to the samples. Final concentrations were expressed as picomoles of lipids per milligram of proteins. Molecular species profiles were constructed using the measured signals of individual species and expressed as picomoles of lipids per milligram of proteins (41).

### β-glucuronidase release and Ca^2+^ response

β-glucuronidase release was determined as described elsewhere (42). To measure Ca^2+^ response, the cells were loaded with Fura-2-AM and probenecid to a final concentration of 1 μM and 2.5 mM, respectively. After 20 min of incubation at 22°C, the cells were washed in BSS-BSA/probenecid and immediately before measurement centrifuged at 805 *g* at 20°C for 5 min, and then resuspended at concentration of 5 × 10^5^/ml in BSS-BSA. Aliquots of 100 μl were inserted into wells of a white 96-well plate. Changes in concentrations of intracellular Ca^2+^ ([Ca^2+^]_i_) were determined by spectrofluorometry using the Infinite 200M plate reader (TECAN) with excitation wavelengths at 340 and 380 nm and with a constant emission at 510 nm. The activator was added manually after 30 s of basal Ca^2+^ measurements. Data are shown as the ratio em510_exc340_/em510_exc380_.

### Flow cytometry analysis

To quantify the surface expression of FcεRI and c-Kit (CD117), mast cells (5 × 10^5^/ml) were exposed simultaneously to anti-mouse FcεRI-FITC and anti-mouse c-Kit-allophycocyanin. After 30 min of incubation on ice, the cells were washed in ice-cold PBS. To quantify the TNF-α production, the cells were preincubated with brefeldin A (5 μg/ml; Santa Cruz Biotechnology) for 2 h, then activated in the presence of brefeldin A (5 μg/ml) with 1 μg/ml of antigen (TNP-BSA) for 1.5 h, centrifuged at 805 *g* for 3 min at 22°C, and fixed with 4% paraformaldehyde dissolved in PBS for 15 min at 22°C. After washing in PBS, cells were permeabilized with 0.1% saponin in PBS for 15 min at 22°C, centrifuged as aforementioned, and resuspended in PBS supplemented with 1% BSA and TNF-α-phycoerythrin for 1 h, followed by washing in PBS. All multicolor flow cytometry data were acquired with BD LSRII and analyzed using FlowJo, version 10 (Becton Dickinson).

### Microscopy

Endogenous expression of ORMDL3, 5-LO, and STIM1 was examined in HMC-1.1 cells, which express high levels of all proteins, and the corresponding antibodies recognize the human epitopes. Cells were allowed to adhere to fibronectin-covered glass for 30 min at 37°C. HMC-1.1 cells nonactivated or activated with 2 μM ionomycin were fixed using 4% paraformaldehyde and subsequently permeabilized with ice-cold 0.1% Triton X-100. The cells were then stained with primary antibodies followed by secondary antibodies. Samples were mounted using 90% glycerol supplemented with 5% *n*-propyl gallate. Images of nonactivated and ionomycin-activated cells were acquired with a confocal laser scanning microscope Leica TCS SP5 equipped with X63/1.40 numerical aperture oil-immersion objective. Alcian blue/safranine or toluidine blue-stained PDMCs were examined in the bright field with a Leica DM6000 fluorescence microscope equipped with a color DFC490 camera and an X63/1.40 numerical aperture oil-immersion objective. Colocalization analysis was performed on set of 30 confocal z-stacks. About 15 for ORMDL + 5-LO combination and 15 for STIM1 + 5-LO combination. For each combination, three time points were used (0, 10, and 30 min) with four to five cells per time point. Each of the obtained z-stacks was deconvolved using a Huygens professional software suite. For the actual colocalization, we used a JACoP plugin for ImageJ. Only Pearson's coefficient was measured using this plugin. Thresholds were set to 0 since images were recorded using a hybrid detector with subsequent software deconvolution as the background was basically 0 intensity.

### Quantitative PCR assessment of mRNA levels

IgE-sensitized BMMCs were activated with antigen (TNP-BSA; 250 ng/ml). One hour later, mRNA was extracted using a MicroElute® Total RNA Kit (VWR; Omega Bio-Tek, Inc). Single-stranded cDNA was synthesized with M-MLV reverse transcriptase (Invitrogen) according to the manufacturer's instructions. Real-time PCR amplifications of cDNAs were performed in 10 μl reaction volumes of a quantitative PCR mix containing 1 M 1,2-propanediol, 0.2 M trehalose, and SYBR green 1 (43) in 384-well plates sealed with LightCycler 480 sealing foil and analyzed by LightCycler 480 (Roche Diagnostics). The following cycling conditions were used: 3 min at 95°C, followed by 50 cycles of 10 s at 95°C, 20 s at 60°C, and 20 s at 72°C. Threshold cycle values were determined by automated threshold analysis of the cycler. The specificity of PCR was evaluated by examining the melting curves. *Ubiquitin* and *Hprt* were used as reference genes, and the expression levels of the studied mRNAs were normalized to the geometric mean of the reference genes of nonactivated control cells. The relative changes in the mRNA expression levels were normalized to the ones of the corresponding controls. The primer sets used for amplification of different cDNA fragments are shown in supplemental Table S1.

### Statistical analysis

Statistical analysis was carried out using the Student's unpaired two-tailed *t* test for comparison of two groups or with nonparametric two-tailed Mann-Whitney test. For comparison of three groups in the experiment, one-way ANOVA with Bonferroni post hoc comparison or nonparametric Kruskal-Wallis test with Dunn's post hoc test was used. Multiple comparisons within the group were tested against the corresponding group control. Calculated *P* values of less than 0.05 were considered significant. For each analysis, the data met the assumptions of the statistical test. Data are expressed as mean ± SEM. All statistical analyses were performed using GraphPad Prism, version 7.3 (GraphPad Software).

## Results

### PDMCs deficient in ORMDL3 exhibit increased antigen-induced calcium mobilization, degranulation response, and FcεRI/IκB-α/TNF-α axis signaling

**Fig. 1:**
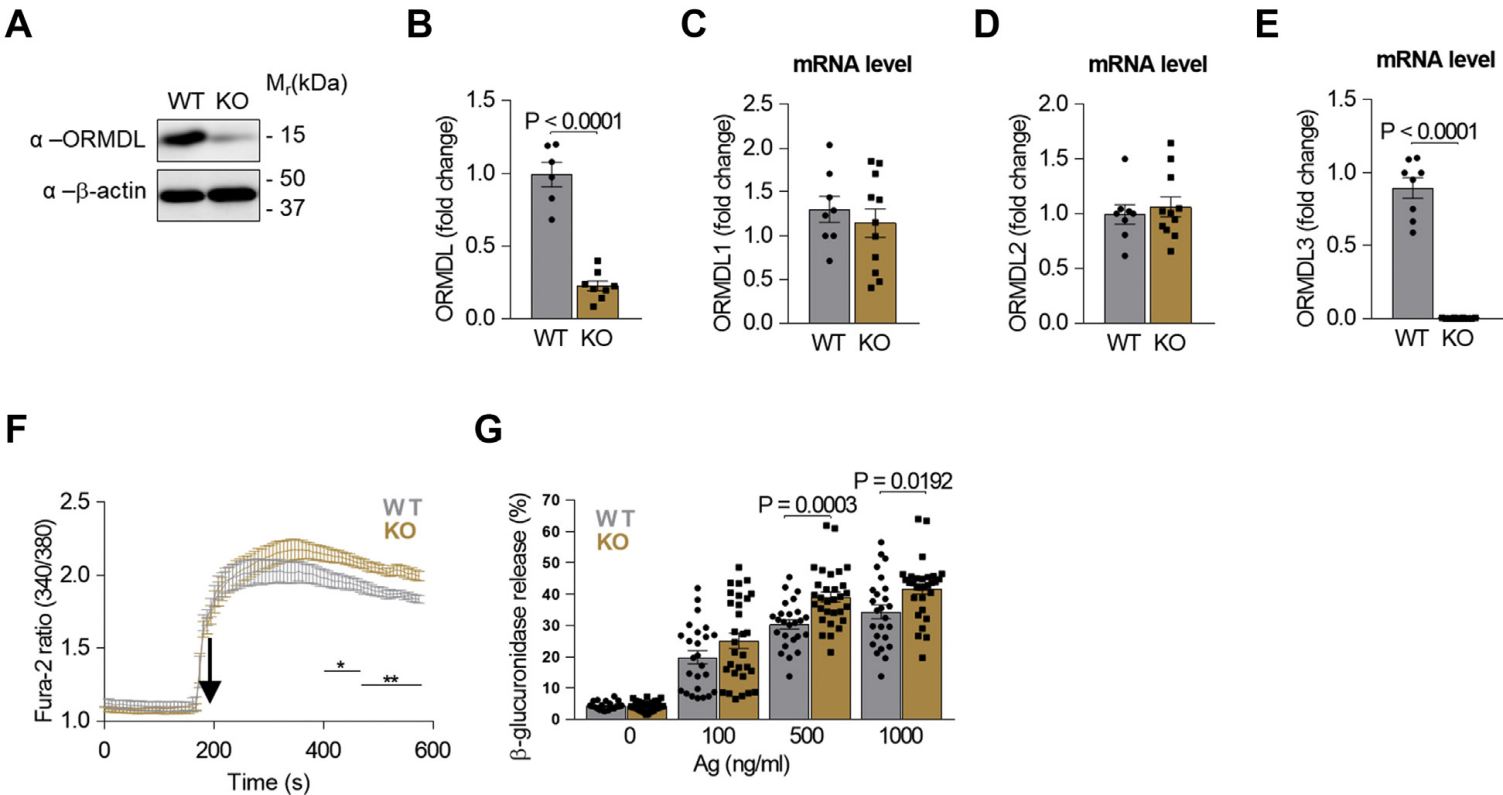
Expression of ORMDL family members and antigen-induced signaling in PDMCs isolated from WT and ORMDL3 KO mice. A: Immunoblot of PDMCs isolated from WT and ORMDL3 KO PDMCs. B: Statistical analysis of global ORMDL family members expression in WT (n = 6) and ORMDL3 KO (n = 8). C–E: RT-qPCR quantification of mRNAs encoding ORMDL1 (C), ORMDL2 (D), and ORMDL3 (E) in resting WT (n = 8) and ORMDL3 KO (n = 11) PDMCs. F: Calcium mobilization in antigen (Ag)-activated PDMCs WT (n = 7) ORMDL3 KO (n = 9). G: Antigen-mediated β-glucuronidase release into supernatants of PDMCs WT (n = 25) and ORMDL3 KO (n = 30) activated for 30 min with various concentrations of antigen. Quantitative data are mean ± SEM, calculated from n, which shows numbers of biological replicates of independently isolated PDMCs. *P* values were determined by unpaired two-sided Student's *t* test.

**Fig. 2:**
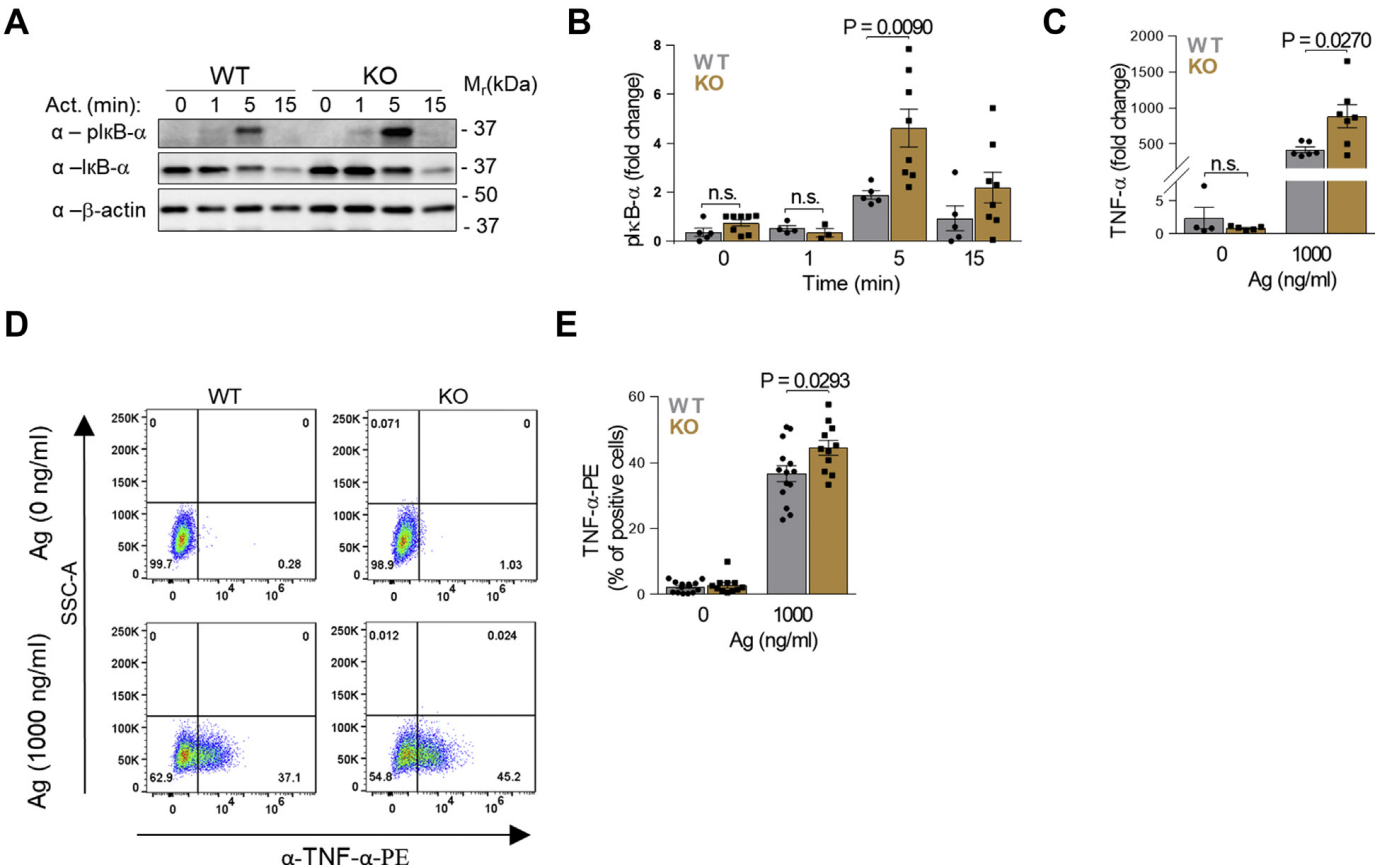
Increased phosphorylation of IκB-α and enhanced expression of TNF-α in PDMCs with ORMDL3 KO. A: IκB-α phosphorylation (pIκB-α) levels were determined by immunoblotting of whole-cell lysates from Ag-activated cells using the corresponding antibody (α). Loading control levels of IκB-α and β-actin were also determined. B: Quantification and statistical evaluation of pIκB-α levels as in (A) normalized to nonactivated WT (n = 5) and ORMDL3 KO (n = 8) PDMCs and corresponding β-actin load. C: RT-qPCR quantfication of mRNAs encoding TNF-α in nonactivated WT (n = 5) and ORMDL3 KO PDMCs (n = 7) and Ag-activated WT (n = 7) and ORMDL3 KO PDMCs (n = 7). D: Flow cytometry analysis of TNF-α production in nonactivated and Ag-activated WT and ORMDL3 KO PDMCs. E: Statistical evaluation of TNF-α data. From the PE positive quadrants as in (D); WT (n = 13) and ORMDL3 KO (n = 11). Quantitative data presented are mean ± SEM, calculated from n, which show numbers of biological replicates of independently isolated PDMCs. *P* values were determined by unpaired two-sided Student's *t* test.

Mast cells respond immediately to external stimuli by releasing preformed proinflammatory mediators stored in secretory lysosomes (44). We have previously reported that BMMCs with ORMDL3 knockdown or concurrently deficient in ORMDL2 and ORMDL3 exhibit increased responsiveness to antigen (29, 30). In contrast, ORMDL3 is a positive regulator in eosinophils (28). These data suggest the unique role of ORMDL3 in mast cells, presumably by engaging other signaling pathways. To corroborate the data and extend these results, we examined the properties of PDMCs isolated from WT and ORMDL3 KO mice prepared with the clustered regularly interspaced short palindromic repeats/CRISPR-associated protein 9 technology (30). PDMCs isolated from both WT and ORMDL3 KO mice exhibited properties of mast cells, including alcian blue/safranin or toluidine blue staining (supplemental Fig. S1A) and comparable expression of the surface FcεRI and c-Kit (CD117; supplemental Fig. S1B, C). ORMDL3 deficiency resulted in ∼77% reduction in ORMDL proteins, as detected by immunoblotting with pan-ORMDL-specific polyclonal serum (29) (Fig. 1A, B). When compared with WT cells, ORMDL3-deficient PDMCs expressed normal levels of ORMDL1 and ORMDL2 transcripts but had an undetectable level of ORMDL3 mRNA (Fig. 1C–E). Antigen-mediated FcεRI triggering in PDMCs with ORMDL3 KO resulted in enhanced calcium mobilization (Fig. 1F) and degranulation measured by β-glucuronidase release (Fig. 1G). ORMDL3-deficient PDMCs also exhibited increased phosphorylation of IκB-α (Fig. 2A, B, supplemental Fig. S2A), which triggers its degradation (45). The levels of IκB-α were in nonactivated cells comparable between WT and ORMDL3-deficient PDMCs (supplemental Fig. S2B). These events were followed by increased mRNA levels of TNF-α (Fig. 2C) as well as production of TNF-α measured by flow cytometry (Fig. 2D, E) in ORMDL3-deficient PDMCs.

### ORMDL3 limits the production of sphingolipids and LTs

**Fig. 3:**
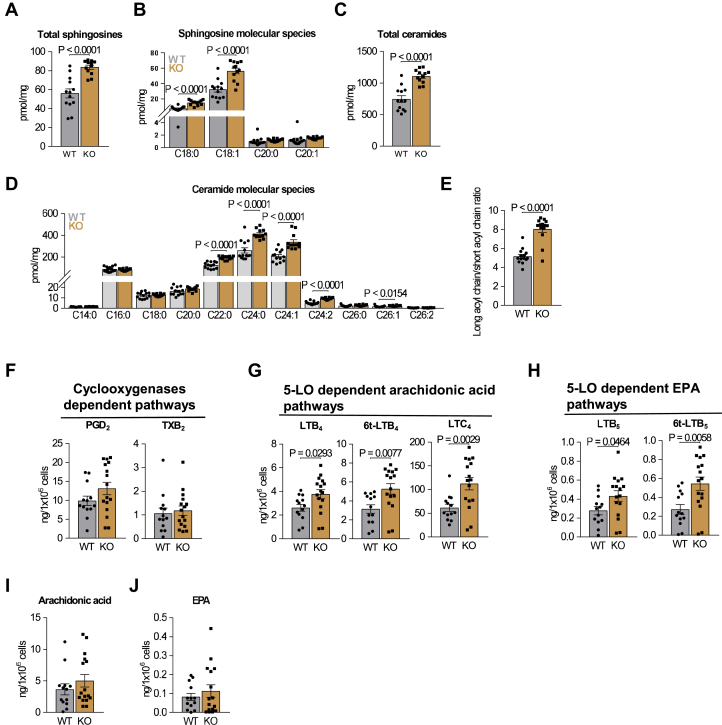
ORMDL3 in PDMCs limits sphingolipid and leukotriene biosynthesis. A–E: LC-ESI-MS/MS analysis of sphingolipids in resting WT (n = 13) and ORMDL3 KO (n = 12) PDMCs. A: The sum of total sphingosines, C18:1, C18:0, C20:1, and C20:0. B: The values of distinct sphingosines C18:1 and C20:1 and sphinganines C18:0 and C20:0. C: The sum of total ceramide fatty acid chain molecular species derived from C18:1 sphingosine. D: Non-2-hydroxylated ceramide molecular species derived from C18:1 sphingosine. E: Ratio of very long acyl chains molecular species (C22:C26) and long acyl chains molecular species (C14:C20) in resting WT (n = 12) and ORMDL3 KO (n = 12) PDMCs. F–J: UPLC MS/MS analysis of eicosanoids from supernatants of antigen-activated PDMCs WT (n = 13) and ORMDL3 KO (n = 16). F: Prostaglandin PGD_2_ and thromboxane TXB_2_ derived from arachidonic acid. G: Leukotrienes derived from arachidonic acid. H: Leukotrienes derived from EPA. I: The total levels of arachidonic acid. J: The total levels of EPA. Quantitative data are mean ± SEM, calculated from n, which show numbers of biological replicates of independently isolated PDMCs. *P* values were determined by unpaired two-sided Student's *t* test.

The regulatory role of ORMDL proteins in sphingolipid synthesis has been described in several cell types (10, 11, 12, 13) including BMMCs (30). Mass spectrometry analysis indicated increased levels of total sphingosines, sphinganine C18:0, and sphingosine C18:1 in PDMCs from ORMDL3 KO (Fig. 3A, B). Total ceramide levels, ceramide molecular species with longer acyl chains (>C22:0), and several 2-hydroxy molecular species were also elevated (Fig. 3C–E and supplemental Fig. S2C).

Mast cells are potent producers of eicosanoids, which include LTs, and prostanoids, divided on prostaglandins and thromboxanes (7, 44). Eicosanoids are formed by conversion of arachidonic acid or EPA substrates and contribute to the duration and magnitude of inflammation, signaling, and wound healing (3, 4, 5, 7, 46). In this study, we examined the role of ORMDL3 in COX and 5-LO-dependent pathways 30 min upon activation with antigen (Fig. 3F–H). The levels of COX-dependent products, PGD_2_ and thromboxane (TX)B_2_, were comparable in PDMCs with ORMDL3 KO and WT (Fig. 3F). However, the production of 5-LO-dependent LTB_4_, 6t-LTB_4_, and LTC_4_ mediators, derived from arachidonic acid (Fig. 3G), or LTB_5_ and 6t-LTB_5_, derived from EPA (Fig. 3H), was increased in ORMDL3-deficient PDMCs. This increase was not associated with changes in 5-LO expression in ORMDL3-deficient cells (supplemental Fig. S2D). The levels of arachidonic acid and EPA were not changed (Fig. 3I, J). The enhanced levels of PGD_2_ in supernatants of 5 h-activated PDMCs with ORMDL3 KO (supplemental Fig. S2E) suggested the contribution of an inducible COX-2, as we showed previously in BMMCs with ORMDL3 knockdown (29).

To examine the effect of inhibitors of sphingolipid synthesis, we used myriocin and fumonisin B_1_ compounds impairing sphingolipid synthesis at distinct levels. Myriocin directly blocks the active site of SPT complex (47), whereas fumonisin B_1_ blocks ceramide synthase (48). We found that both drugs inhibited LTB_4_ and PGD_2_ production in PDMCs comparably with ORMDL3 KO or WT (supplemental Fig. S2F, G). The levels of arachidonic acid were also decreased (supplemental Fig. S2H).

The combined data suggest that ORMDL3 limits the LT pathway in the early stages of mast cell activation and PGD_2_ production in later stages after FcεRI triggering.

### 5-LO interacts with ORMDL3 and the SPT complex

**Fig. 4:**
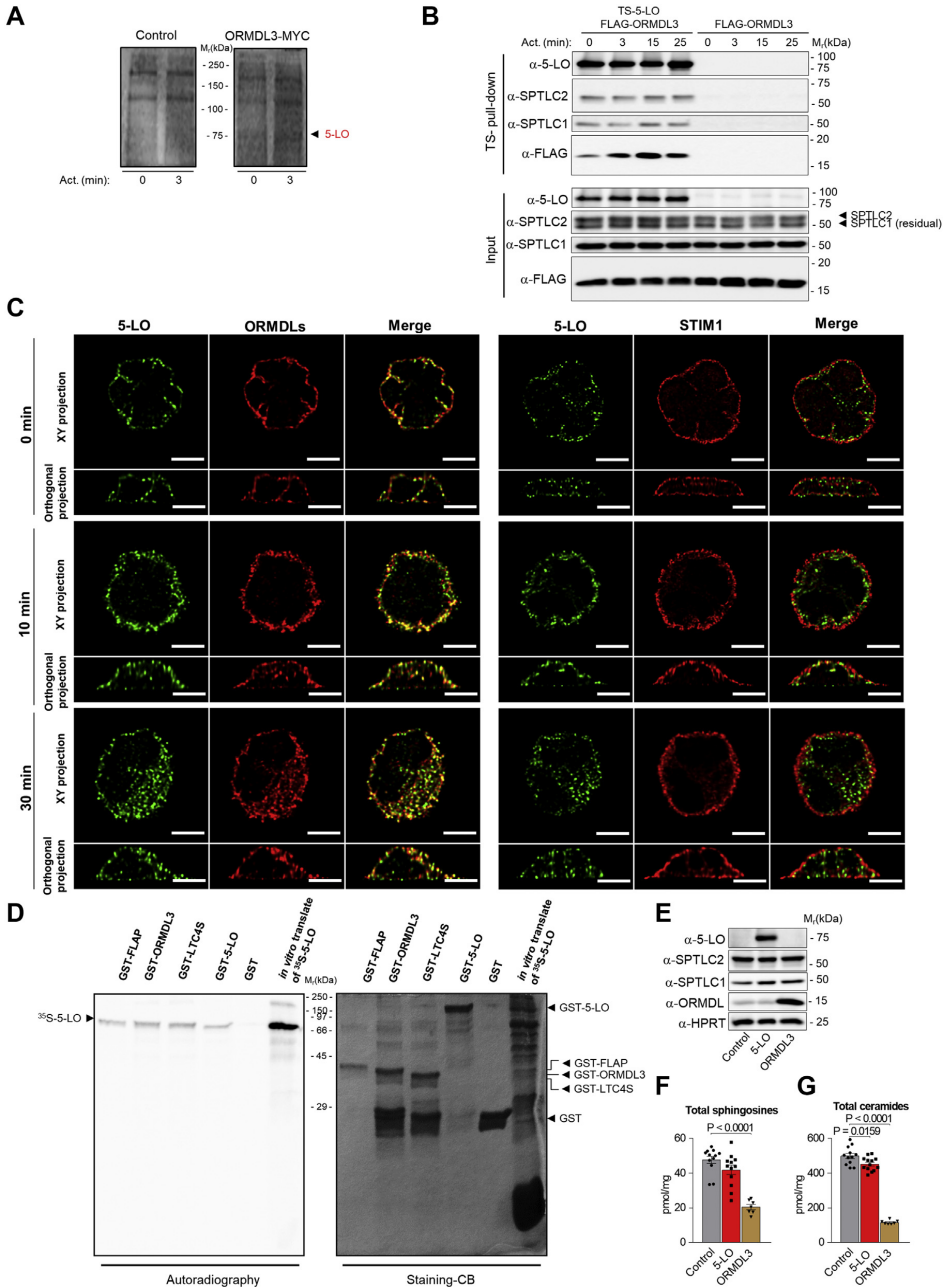
Identification of the ORMDL3/5-LO/SPTLC1/SPTLC2 complex. A: ORMDL3-MYC immunoprecipitated from lysates of BMMCL expressing ORMDL3-MYC or empty vectors (control) and activated or not with thapsigargin. The immunoprecipitates were size-fractionated by SDS-PAGE and stained by Coomassie blue. ←5-LO indicates position of 5-LO as identified by mass spectrometry. B: 5-LO interacts with ORMDL3, SPTLC1, and SPTLC2. Twin-Strep-tag (TS) affinity purification from lysates of HEK293FT cells stably transduced with FLAG-ORMDL3 alone or in combination with TS-5-LO and activated for 0, 3, 15, or 25 min with thapsigargin. The proteins were analyzed by immunoblotting using the corresponding antibodies. C: Colocalization of endogenous 5-LO with ORMDLs (left panel) or STIM1 (right panel) in nonactivated (0 min) or activated HMC-1.1 cells for 10 or 30 min with ionomycin. Scale bars represent 5 μm. D: Physical interaction of 5-LO with ORMDL3. FLAP, ORMDL3, LTC4S, and 5-LO were tagged with GST and expressed in bacteria. GST-tagged proteins and GST alone were purified on glutathione-coated beads in the presence of in vitro translate expressing ^35^S-5-LO. SDS-PAGE followed by autoradiography was used to detect ^35^S-5-LO. The gel was stained with Coomassie brilliant blue (CB staining) to determine the loading levels of GST and GST-tagged proteins. E: SDS-PAGE of lysates from HEK293 cells stably transduced with empty vector (control), murine 5-LO, or murine ORMDL3 developed with the indicated protein-specific antibodies. F and G: LC-ESI-MS/MS analysis of sphingolipids in resting HEK293 cells stably transduced with murine 5-LO (n = 13), murine ORMDL3 (n = 7), or empty vector (control, n = 12). F: Total sphingosines, the sum of C18:1 and C18:0 is calculated. G: The sum of total ceramide fatty acid chain molecular species (including 2-hydroxy ceramide molecular species), derived from d18:1 sphingosine, was calculated. Data in B and D are representative of three independent experiments. Quantitative data in F and G are mean ± SEM, calculated from n, which show numbers of biological replicates of independently transduced cells. *P* values were determined by one-way ANOVA with Bonferoni post hoc test.

To identify new interaction partners of ORMDL3, which could explain its association with various inflammatory diseases, and to study the unique functions of ORMDL3 in mast cells, we performed coimmunoprecipitation experiments using mast cells. For these experiments, we transduced cells of BMMCL with ORMDL3-MYC or empty vector, used as a control, and activated or not with thapsigargin, an inhibitor of the SERCA pump. A band of about 75 kDa visible only in the activated ORMDL3-MYC transfected cells (Fig. 4A) was analyzed by mass spectrometry and determined to be 5-LO (GenBank: AAC37673.1). The finding that ORMDL3 interacts with 5-LO was confirmed in experiments with the HEK293FT cell line not expressing endogenous 5-LO. HEK293FT cells stably transduced with FLAG-ORMDL3 alone or together with TS-5-LO were activated for various time intervals with thapsigargin. TS-5-LO from postnuclear supernatants was isolated by affinity purification on Strep-Tactin-coated resin (Fig. 4B). We found that ORMDL3 is associated with TS-5-LO in resting HEK293FT cells, and this association is increased after activation (Fig. 4B). Since FLAG-tagged ORMDL3 interacts with SPTLC1 in HEK293T cells (10), we used the TS-5-LO pull-down samples to determine whether 5-LO forms complexes with the subunits of SPT. We found that 5-LO was associated with both SPTLC1 and SPTLC2 subunits of SPT (Fig. 4B). To analyze the localization of endogenous ORMDL proteins and 5-LO by microscopy, we used HMC-1.1, which express both proteins, ORMDL and 5-LO. For this study, we used our own antibody (29) recognizing ORMDLs, as determined by control experiments (supplemental Fig. S3A–C). We found that endogenous 5-LO forms spots that overlap with ORMDL domains, both in nonactivated and activated cells (Fig. 4C, left panel). The adherence of HMC-1.1 cells to fibronectin could trigger integrin signaling and explain the association of 5-LO with ER even in nonactivated cells. In contrast, 5-LO is not present in domains with STIM1, another ER-resident protein crucial for mast cell signaling (34, 45) (Fig. 4C, right panel). The Pearson's coefficients of correlation between endogenously expressed 5-LO and ORMDLs and between 5-LO and STIM1 at various time intervals after cell activation are shown in supplemental Fig. S4A. In nonactivated BMMCL, 5-LO is also present in the cytoplasm, and after activation was increasingly associated with membrane fractions and moved into the nuclear pellet fraction (supplemental Fig. S4B, C). Next, we examined whether ORMDL3 directly interacts with 5-LO. To this end, we used GST pull-down assay using bacterial lysates expressing GST constructs combined with radioactively labeled ^35^S-5-LO generated by in vitro translation. We found that ^35^S-5-LO binds directly to GST-ORMDL3, similarly to positive controls FLAP (49, 50), LTC4S (50), and 5-LO itself, resulting in its dimerization (51), but not to GST alone (Fig. 4D). To corroborate these results, we performed a reverse GST pull-down assay with radioactively labeled ^35^S-ORMDL3 (supplemental Fig. S5A). We also examined the GST pulldowns in which we combined the BMMC lysates containing endogenous levels of 5-LO, ORMDL3, and SPTCL1 and found clear coprecipitation. When STIM1 was examined in the assay, no coprecipitation was observed (supplemental Fig. S5B).

To evaluate the functional link between 5-LO and the SPT complex, we transduced HEK293FT cells with murine 5-LO, ORMDL3, or empty vector (Fig. 4E) and examined the levels of total sphingosines and ceramides. As expected, increased expression of ORMDL3 impaired the total level of sphingosines and ceramides (Fig. 4F, G). Importantly, we found that exogenously expressed 5-LO also reduced total ceramide levels (Fig. 4G). Detail analysis showed that 5-LO expressed in HEK293FT cells decreased the levels of ceramides C16:0 and C24:0 (supplemental Fig. S6A). Overexpressed ORMDL3 as the regulator of the SPT complex attenuated almost all examined ceramides (supplemental Fig. S6A). ORMDL3 overexpression not only decreased the total ceramide levels but also caused changes in the ratio of the remaining ceramides in favor of the shorter acyl chain length (supplemental Fig. S6B).

HEK293 cells do not express components of LT synthesis. To examine the effect of FLAP-dependent 5-LO anchoring to the ER membrane on sphingolipid production, we transfected 5-LO and FLAP genes individually or together into HEK293 cells (supplemental Fig. S6C) and analyzed sphingolipids. We found that sphingosines were not significantly changed in any group tested (supplemental Fig. S6D). When ceramides were analyzed, 5-LO significantly affected, when compared with FLAP alone, the production of ceramides. However, when FLAP and 5-LO were transfected simultaneously, the production of ceramides was increased when compared with 5-LO-alone transfected cells (supplemental Fig. S6E). Based on these data, we propose that FLAP is located in domains that are not involved in sphingolipid production. When FLAP and 5-LO are coexpressed, 5-LO binds preferentially to FLAP domains, and therefore, its inhibitory effect on ceramides is not executed; this could lead to increased production of ceramides in 5-LO and FLAP cotransfected cells (supplemental Fig. S6E).

### Impaired binding of 5-LOW^13/75/102A^ mutant to the SPT complex and ORMDL3

**Fig. 5:**
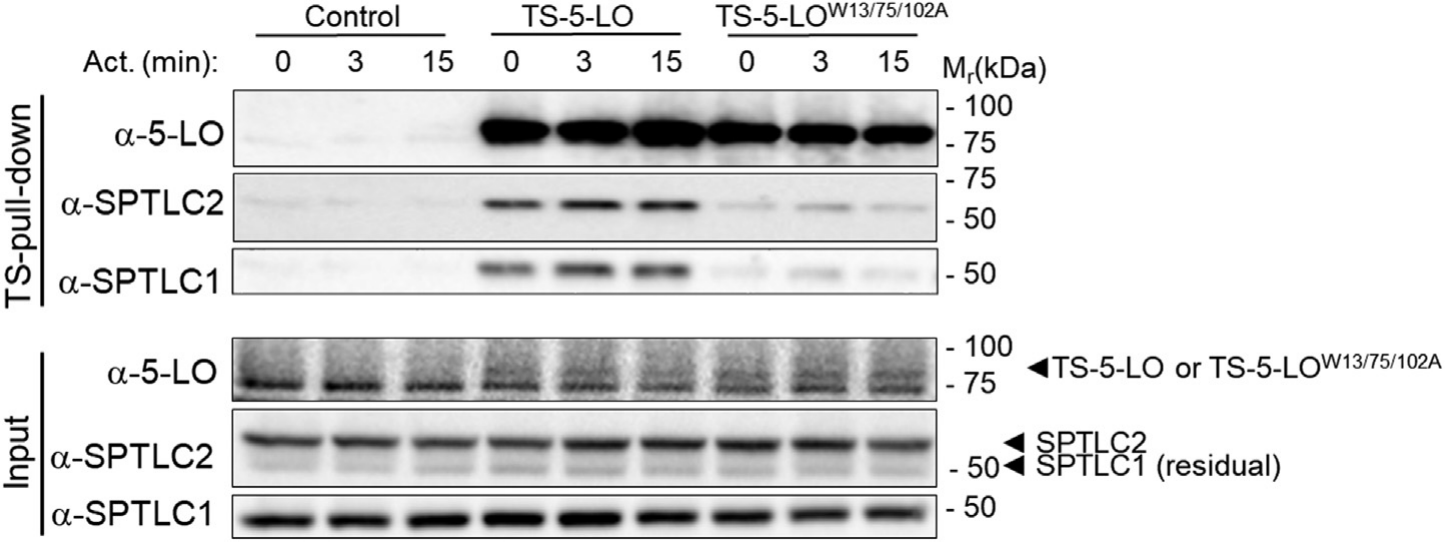
Impaired binding of TS-5-LO^W13/W75/W102A^ with the SPT complex. TS-5-LO and TS-5-LO^W13/W75/W102A^ were pulled down from Ag-activated BMMCL, size-fractionated by SDS-PAGE, and analyzed by immunoblotting with corresponding antibodies (α-). The input of the analyzed proteins is also shown. Data are representative of three independent experiments.

To identify the 5-LO domain involved in the SPT complex binding, we used a triple Trp mutant of 5-LO^W13/75/102A^ deficient in binding to phosphatidylcholine-enriched membranes (52) and coactosin-like protein (53) via the C2-like domain. Pull-down assays from postnuclear lysates of BMMCL stably expressing TS-5-LO and the mutant variant TS-5-LO^W13/75/102A^ showed that the interaction of TS-5-LO^W13/75/102A^ with SPTLC1 and SPTLC2 was impaired in resting and antigen-activated cells (Fig. 5). These data suggested that the C2-like domain of 5-LO is involved in the association of 5-LO with the SPT complex. The quantification of the SPTLC1 and SPTLC2 levels as well as the normalization of SPTLC1 and SPTLC2 pull down relative to their expression levels in the lysates is shown (supplemental Fig. S7A–D). Similarly, HA-ORMDL3, SPTLC1, and SPTLC2 exhibited reduced interaction with TS-5-LO^W13/75/102A^ in HEK293FT cells (supplemental Fig. S7E).

To verify the specificity of these interactions, we performed pull down from postnuclear lysates of HEK293FT cells transduced with FLAG-ORMDL3 alone or together with TS-5-LO, TS-5-LO^W13/75/102A^, or streptactin-tagged truncated version of STIM1 (TS-tSTIM1). We found that endogenously expressed STIM1 is not coprecipitated with the streptactin-tagged proteins (supplemental Fig. S7F).

### The crosstalk between sphingolipid and eicosanoid metabolic pathways

**Fig. 6:**
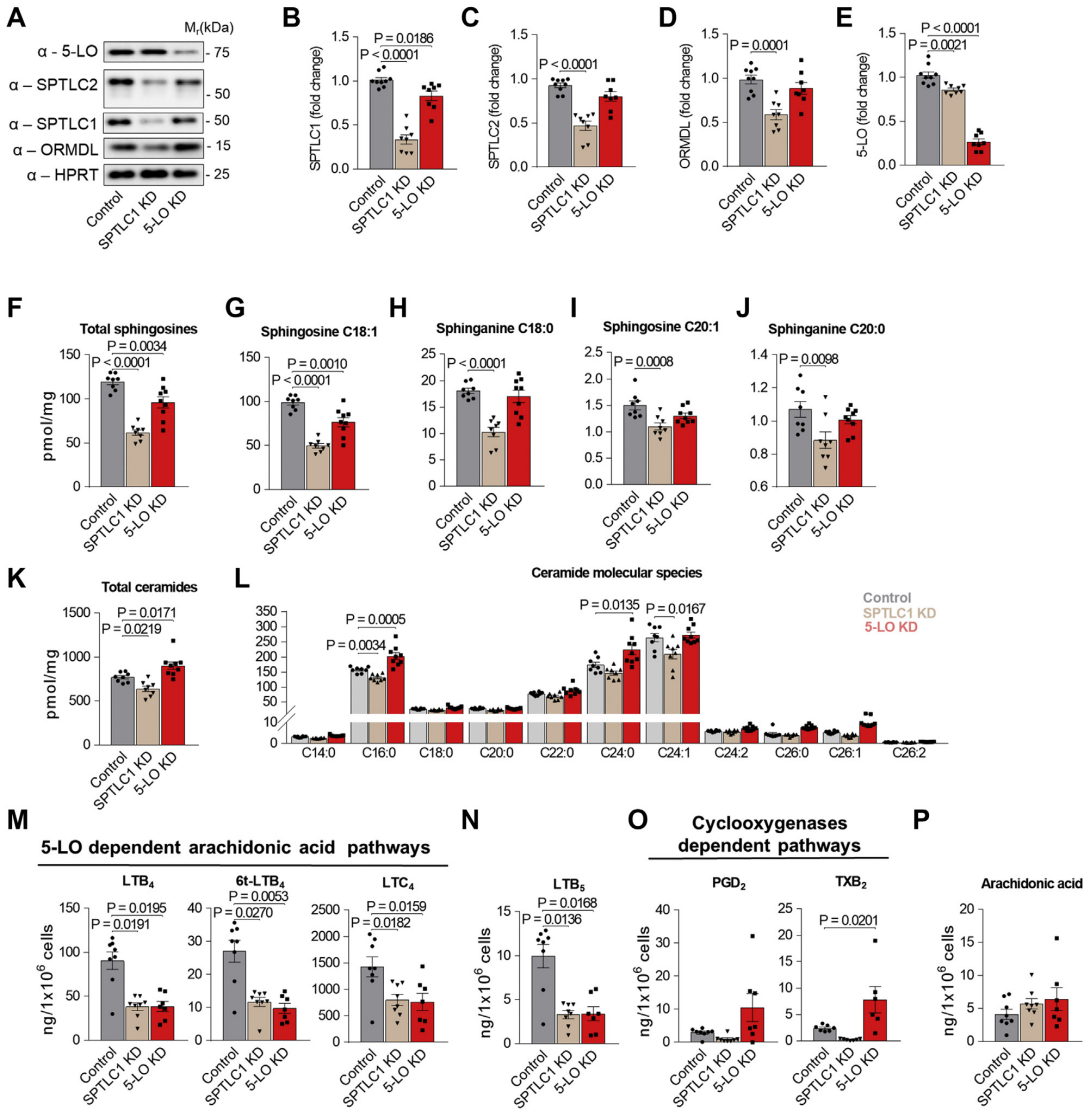
Changes in the metabolism of sphingolipids and eicosanoids in BMMCL with SPTLC1 or 5-LO knockdowns (KDs). A: Lysates from BMMCL transduced with empty vector (control), SPTLC1 shRNA (SPTLC1 KD), and 5-LO shRNA (5-LO KD) were assessed by immunoblotting with the indicated antibodies. B–E: Quantification of data as in A, normalized to expression in controls and HPRT load. Controls (n = 9), SPTLC1 KD (n = 8), and 5-LO KD (n = 8). F–L: LC-ESI-MS/MS analysis of sphingolipids in Ag-activated transduced BMMCL: control (n = 8), SPTLC1 KD (n = 8), and 5-LO KD (n = 9). F: The sum of total sphingosines, C18:1, C18:0, C20:1, and C20:0, is calculated. G–J, The values of distinct sphingosines C18:1 and C20:1 and sphinganines C18:0 and C20:0 are shown. K: The sum of total ceramide fatty acid chain molecular species, derived from C18:1 sphingosine. L: Non-2-hydroxylated ceramide molecular species derived from C18:1 sphingosine. M–P: UPLC MS/MS analysis of eicosanoids from supernatants of Ag-activated BMMCL: controls (n = 8; except TXB_2_ n = 7), SPTLC1 KD (n = 8; except TXB_2_ n = 7), and 5-LO KD (n = 7; except TXB_2_ n = 6). Quantitative data are mean ± SEM, calculated from n, which show numbers of biological replicates of independently transduced cells. *P* values were determined by one-way ANOVA with Bonferroni post hoc test except for LTB_4_, 6t-LTB_4_, and LTB_5_ data, which were compared by nonparametric Kruskal-Wallis test with Dunn's post hoc test.

The finding that ORMDL3 deficiency increases the conversion of arachidonic acid to LTs and that 5-LO interacts with ORMDL3, SPTLC1, and SPTLC2 led us to examine the functional crosstalk between the 5-LO- and SPT complex-dependent metabolic pathways. We used BMMCL with knockdown of 5-LO or SPTLC1 (Fig. 6A and supplemental Fig. S8A). SPTLC1 knockdown decreased not only the expression of SPTLC1 (∼66%) but also that of SPTLC2 (∼53%), ORMDLs (∼41%), and 5-LO (∼14%). In contrast, 5-LO knockdown only lowered the expression of 5-LO (∼73%) and SPTLC1 (∼17%) but had no effect on SPTLC2 and ORMDL expression (Fig. 6A–E and supplemental Fig. S8A). Cells with SPTLC1 knockdown exhibited lower levels of total sphingosines (Fig. 6F), including de novo-formed products dependent on the enzymatic activity of the SPT complex, sphinganines (dihydrosphingosines) C18:0 and C20:0, as well as sphingosines C18:1 and C20:1 (Fig. 6G–J) resulting from de novo synthesis and ceramide degradation, respectively (6). Cells with 5-LO knockdown showed a decrease in total sphingosines (Fig. 6F). Interestingly, only sphingosine C18:1 was significantly reduced but not sphingosine C20:1 and de novo-formed sphinganines C18:0 and C20:0 (Fig. 6G–J). These data suggest that ceramides are less degraded in BMMCL with 5-LO knockdown. Indeed, the total ceramide levels were enhanced in cells with reduced 5-LO expression (Fig. 6K). The enhanced levels of molecular species C16:0, C24:0, and C24:1-OH in cells with 5-LO knockdown (Fig. 6L and supplemental Fig. S8B) are in accord with the results obtained in HEK293FT cells with overexpressed 5-LO (supplemental Fig. S6A). The mild inhibitory effect of SPTLC1 knockdown on ceramide synthesis can be explained by the retained expression ratio between the SPT complex and ORMDL3 (Fig. 6A–D). In antigen-activated 5-LO knockdown cells, the formation of LTs was decreased (Fig. 6M, N), but COX-dependent product TXB_2_ was increased, as the arachidonic acid substrate is less converted to LTs (Fig. 6O). In line with previous findings, the production of LTs in cells with SPTLC1 knockdown was decreased to the levels seen in 5-LO knockdown (Fig. 6M, N). Although 5-LO expression is lowered in SPTLC1 knockdown (Fig. 6E), the competition of COXs with 5-LO for arachidonic acid substrate was not observed, as was the case in the 5-LO knockdown (Fig. 6O). Furthermore, the levels of arachidonic acid in cells with SPTLC1 knockdown were comparable with the levels in control cells. In contrast, inhibitors of sphingolipid synthesis, myriocin, and fumonisin B_1_ decreased the release of arachidonic acid when compared with control cells (supplemental Fig. S2H), suggesting an additional level of regulation in these cells.

## Discussion

In our study, we used PDMCs from mice with ORMDL3 KO and cell lines with enhanced or reduced expression of ORMDL3, 5-LO, or SPTLC1 to determine the role of these proteins in the metabolic pathways leading to the production of sphingolipids and eicosanoids. In initial experiments, we found that the absence of ORMDL3 in PDMCs resulted in increased antigen-induced calcium response, degranulation, IκB-α/TNF-α axis activity, and COX2-dependent PGD_2_ production. Antigen-activated PDMCs with ORMDL3 KO exhibited increased production of LTs (LTB_4_, 6t-LTB_4_, LTC_4_, LTB_5_, and 6t-LTB_5_), suggesting an inhibitory role of ORMDL3 in the 5-LO metabolic pathway after FcεRI triggering. This conclusion was extended by the finding showing that both ORMDL3 and the subunits of SPT complex interact with 5-LO, a rate-limiting enzyme in LT synthesis.

The connection between sphingolipid and eicosanoid pathways is linked to the biological activity of their mediators. Thus, the nonvesicular C1P transfer regulates phospholipase A2 (PLA2)-dependent release of arachidonic acid (54), and the second messenger S1P induces expression of COX-2 (55). These data are in concordance with our previous findings that the deficiency of ORMDL2 and ORMDL3 in BMMCs causes increased S1P production (30) and that BMMCs with ORMDL3 knockdown cells exhibit increased translocation of the p65 subunit of NF-κB into nuclei followed by increased cytokine secretion and COX2-dependent PGD_2_ synthesis (29). Here, we report that ORMDL3 interacts with 5-LO. These data were supported by the finding that another ER-membrane resident protein, STIM1, was not associated in the pull downs of TS-5-LO, GST-5-LO, or GST-ORMDL3. Moreover, the TS-5-LO^W13/75/102A^ mutant form, which exhibit impaired binding of 5-LO to the phosphatidylcholine-enriched membranes (52) and coactosin-like protein (53), abolishes the interaction with ORMDL3, SPTLC1, and SPTLC2. Thus, the lipid environment plays an important role in this interaction. The colocalization study showed that endogenously expressed ORMDL3 and 5-LO occupy overlapping domains on the ER membrane. It should be noted that another ER-membrane localized protein, STIM1, occupied different domains in the ER membrane, supporting the biological relevance of ORMDL3-5-LO crosstalk. In contrast to the ubiquitously expressed SPT complex, the expression of 5-LO is mainly constrained to various leukocytes (56), and therefore, the ORMDL3/SPT/5-LO complex could be naturally formed only in these cells. As indicated by BioGPS (probeset 1441962), the 5-LO coding gene (*Alox5*) in mice is predominantly expressed in mast cells and only in smaller amounts in some other cell types. Moreover, Dwyer *et al.* (57) showed that the expression of *Alox5* substantially differs in various murine mast-cell subsets. *Alox5* expression was found mostly in mast cells isolated from peritoneum, followed by mast cell subsets isolated from the trachea and esophagus. The lowest expression of *Alox5* was determined in mast cells isolated from the tongue and skin tissue. Thus, PDMCs used in this study belong to the cell types expressing components of both metabolic pathways at high levels, which allowed us to use various techniques to manipulate the composition of the ORMDL3/SPT/5-LO complex. Further experiments would be required to determine the role of 5-LO in other cell types and mast cell subsets.

Removal of ORMDL3 resulted in enhanced production of sphingolipids, which is in accord with the results obtained with lung epithelial A549 cells (58) or BMMCs deficient in ORMDL3 (30). In contrast, in HEK293T and HeLa cell lines, it was necessary to silence all ORMDL members to get increased levels of ceramides (10, 13).

Mass spectrometry analysis of lipids showed that the reduced production of LTs in cells with 5-LO knockdown was accompanied by enhanced production of TXB_2_. This observation could be explained by the competition of 5-LO with COXs for arachidonic acid as a substrate. The balance between COX and 5-LO enzymatic activity was described in macrophages lacking FLAP, which failed to support 5-LO-dependent production of LTC_4_ and instead, increased the yields of COX-dependent products PGE_2_ and TXB_2_ (59). Interestingly, the reduced production of LTs in mast cells with SPTLC1 knockdown, as observed in this study, was not compensated for by the enhanced production of PGD_2_ and TXB_2_. We also found that 5-LO expression inversely correlated with ceramide levels. Our finding of reduced levels of sphingosine C18:1, an intermediate of a degradative salvage pathway, and accumulation of ceramides in cells with 5-LO knockdown suggested that 5-LO is involved in the ceramide turnover independently of de novo sphinganine formation. The depletion and silencing of ORMDL3 in BMMCs and SPTLC1 in BMMCL, respectively, had no significant effect on the levels of arachidonic acid, suggesting that PLA2 activity is not changed in these cells. In contrast, mycotoxins myriocin and fumonisin B_1_, which inhibit the sphingolipid biosynthesis (47, 48), decreased the release of arachidonic acid and subsequently production of LTB_4_ and PGD_2_ in WT and ORMDL3 KO BMMCs. It was shown that fumonisin B_1_ blunts the secretory PLA2-dependent release of arachidonic acid in P388D_1_ macrophages (39), and FTY720, an immunosuppressive agent derived from myriocin, selectively suppresses the cytosolic cPLA2 activity (60). We assume that the locally formed products in the pathway crosstalk form a microenvironment affecting the yields of metabolites; however, we cannot exclude that the physical interference also plays a role in this process, presumably as a negative feedback loop to suppress the 5-LO activity in antigen-activated cells. The role of TNF-α in the regulation of LT synthesis should also be considered (61). However, in our study, the enhanced LT synthesis preceded the increased intracellular levels of TNF-α.

The function of mast cells was recently examined in mice with locally silenced ORMDL3 (29, 62) or in mice deficient in ORMDL2, ORMDL3, or both proteins (30). Passive cutaneous anaphylaxis was enhanced in mice with intradermally silenced ORMDL3 (29, 62) or in double-deficient ORMDL2 and ORMDL3 KO mice (30). We assume that the increased passive cutaneous anaphylaxis in such mice is a result of increased mast cell responsiveness to which metabolites of sphingolipid and eicosanoid pathways are contributors (59, 63).

The tight regulation of ORMDL expression is important in maintaining sphingolipid homeostasis. In this study, we also found that the knockdown of SPTLC1 (∼66%) in mast cells was associated with decreased expression of SPTLC2 (∼53%) and ORMDLs (∼41%), suggesting a regulatory feedback acting to maintain a constant ratio between the proteins involved in sphingolipid homeostasis. This regulatory feedback was also observed in the opposite direction, where overexpression of SPT complex enhanced expression of ORMDLs (64). These data are in accord with the finding that silencing of all SPTLCs in the human liver Huh7 cell line resulted in modified transcription of numerous genes involved in lipid metabolism (65). Previous studies reported that antigen-mediated activation could also change the expression levels of ORMDL3. Thus, triggering of FcεRI with multivalent antigen decreased the ORMDL3 levels in mast cells (29, 62), whereas challenge with allergen increased the levels of ORMDL3 in mouse bronchial epithelial cells, bone marrow-derived eosinophils, or lung macrophages (16). Treatment of macrophage cell line RAW267.4 or bone marrow-derived macrophages with lipopolysaccharide for 4 h decreased ORMDL expression, whereas sphinganine production was increased (12, 66). It should be noted that overexpression of gasdermin B (*GSDMB*) in human bronchial epithelial cells increased 5-LO expression (67). *GSDMB*, which similarly to *ORMDL3* maps into the 17q21 region, exhibits associated allelic expression with *ORMDL3* in lymphoblastoid cell lines (23) and in the human primary CD4^+^ T cells and CD8^+^ T cells (31). Thus, both proteins, ORMDL3 and GSDMB, are involved in the regulation of 5-LO-dependent pathway.

**Fig. 7:**
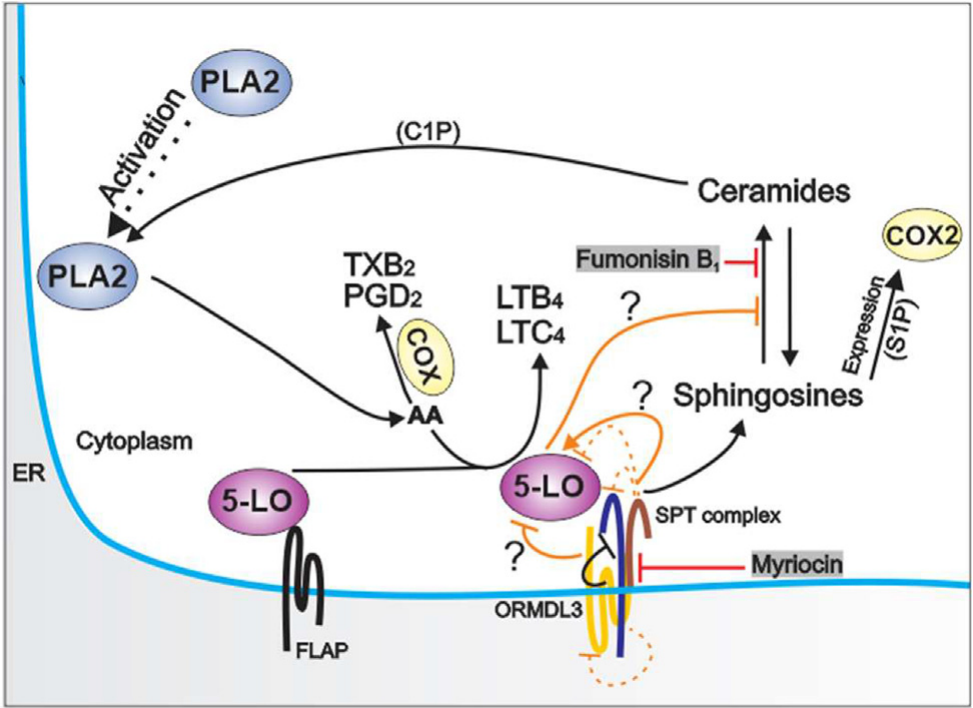
Scheme of the crosstalk of sphingolipid and eicosanoid metabolic pathways. The sphingolipid pathway is known to involve the SPT complex, which is negatively regulated by ORMDL family proteins. This pathway leads to the production of sphingosines and ceramides. The eicosanoid pathway involves PLA2 and 5-LO. When activated, PLA2 releases arachidonic acid (AA) from ER membranes, which is utilized by 5-LO and cyclooxygenases (COX) as a substrate for the production of precursors of leukotrienes and prostaglandins, respectively. The connection between eicosanoid and sphingolipid pathways is linked to the activity of their mediators. It has been shown that the nonvesicular C1P transfer regulates PLA2-dependent release of arachidonic acid (54) and that second messenger S1P induces expression of cyclooxygenase 2 (COX2) (55). Data in this study indicate that ORMDL3 interacts with 5-LO and that 5-LO interacts with SPT complex subunits, SPTLC1 and SPTLC2. The functional consequences of the leukotriene and sphingolipid pathway crosstalk are shown (in orange) and involve *1*) inhibitory role of ORMDL3 on the activity of 5-LO and eicosanoid production; *2*) activatory role of SPTLC1 on 5-LO activity; *3*) inhibitory effect of 5-LO on ceramide levels. Moreover, reduced SPTLC1 levels are followed by decreased expression of SPTLC2 and ORMDL3 (dashed lines). Sphingolipid synthesis inhibitors, myriocin and fumonisin B_1_, which block the activity of SPT complex and ceramide synthase, respectively, affect the release of AA from membranes and subsequently the formation of LTB_4_ or PGD_2_. These data are in line with the regulatory roles of ceramides on the PLA2 activity (39, 54). Thus, the crosstalk between eicosanoid and sphingolipid pathways seems to be mediated via metabolic mediators (ceramides, S1P, and C1P) and physical interaction of ORMDL-SPT complex and 5-LO.

The emerging literature data indicate that the expression levels of ORMDL3 play an important role in the balance between the healthy state and distinct inflammatory disorders (17, 18, 20, 21, 22, 23). Our data suggest a close association between the initial steps of LT and sphingolipid biosynthesis based on the cell type-dependent ability to express enzymes of both lipid pathways simultaneously (Fig. 7). Since dysregulation of sphingolipid and eicosanoid metabolism is involved in inflammatory processes, the physical and functional crosstalk of both lipid pathways with ORMDL3 extends our understanding of the ORMDL3 function and its association with various diseases. We propose that changes in the composition of the ORMDL3/SPT/5-LO complex have a profound effect on the production of LT signaling mediators and sphingolipid metabolism and in this way could contribute to the duration and magnitude of inflammation.

## Data availability

Peptide raw mass spectrometry data are accessible in the public repository: https://figshare.com/s/cd753611ee2d959ee5d0. Peptide mass fingerprints are included in the supplemental data.

## Supplemental data

This article contains supplemental data (29, 52).

## Conflict of interest

The authors declare that they have no conflicts of interest with the contents of this article.
