# Supplementary material for: Crosstalk between ORMDL3, serine palmitoyltransferase, and 5-lipoxygenase in the sphingolipid and eicosanoid metabolic pathways
Source: J Lipid Res. 2021 Sep 22;62:100121. doi: 10.1016/j.jlr.2021.100121 (PMC8527048; doi:10.1016/j.jlr.2021.100121)
Supplement: Supplemental data 1 [file mmc2.pdf]

MATRIX SCIENCE Mascot Search Results

```
User            : q
Email           :
Search title    : Bugajev_101118_0_D23_000002.d
MS data file    : D23.mgf
Database        : Uniprot 20210818 (565254 sequences; 203850821 residues)
Taxonomy        : Mammalia (mammals) (67385 sequences)
Timestamp       : 18 Aug 2021 at 14:39:43 GMT
Top Score       : 149 for Mixture 1, LOX5_MOUSE + K1C10_HUMAN + K2C1_HUMAN
```

	Uniprot	Decoy
Protein hits above identity threshold	4	0
Highest scoring protein hit	81	26

Mascot Score Histogram

Protein score is -10*Log(P), where P is the probability that the observed match is a random event.
Protein scores greater than 61 are significant (p<0.05).

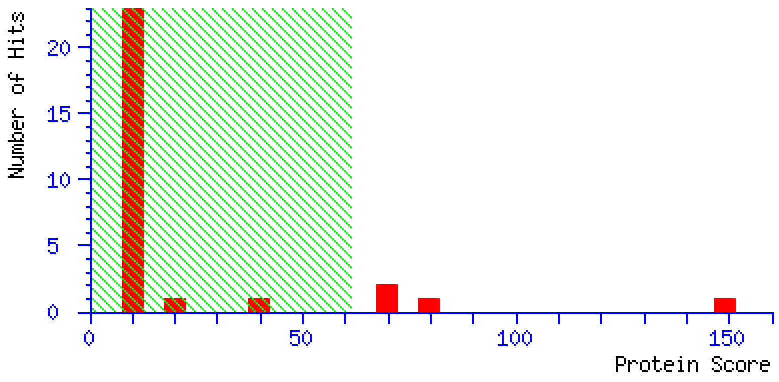

Concise Protein Summary Report

Format As	Concise Protein Summary	Help
	Significance threshold p< 0.05	Max. number of hits AUTO

Re-Search All | Search Unmatched

```
1.    Mixture 1    Total score: 149    Expect: 8.5e-011  Matches: 56
      Components (only one family member shown for each component):
```

LOX5_MOUSE **Mass:** 78602 **Score:** 81 **Expect:** 0.00049 **Matches:** 21
Polyunsaturated fatty acid 5-lipoxygenase OS=Mus musculus OX=10090 GN=Alox5 PE=1 SV=3
K1C10_HUMAN **Mass:** 59020 **Score:** 70 **Expect:** 0.0061 **Matches:** 18
Keratin, type I cytoskeletal 10 OS=Homo sapiens OX=9606 GN=KRT10 PE=1 SV=6
K2C1_HUMAN **Mass:** 66170 **Score:** 69 **Expect:** 0.0089 **Matches:** 18
Keratin, type II cytoskeletal 1 OS=Homo sapiens OX=9606 GN=KRT1 PE=1 SV=6

---

2. LOX5_MOUSE **Mass:** 78602 **Score:** 81 **Expect:** 0.00049 **Matches:** 21
Polyunsaturated fatty acid 5-lipoxygenase OS=Mus musculus OX=10090 GN=Alox5 PE=1 SV=3
LOX5_MESAU **Mass:** 78565 **Score:** 44 **Expect:** 3 **Matches:** 15
Polyunsaturated fatty acid 5-lipoxygenase OS=Mesocricetus auratus OX=10036 GN=ALOX5 PE=2 SV=2
LOX5_RAT **Mass:** 78779 **Score:** 37 **Expect:** 12 **Matches:** 15
Polyunsaturated fatty acid 5-lipoxygenase OS=Rattus norvegicus OX=10116 GN=Alox5 PE=1 SV=3
LOX5_HUMAN **Mass:** 78675 **Score:** 10 **Expect:** 6.4e+003 **Matches:** 9
Polyunsaturated fatty acid 5-lipoxygenase OS=Homo sapiens OX=9606 GN=ALOX5 PE=1 SV=2
LYSC_FELCA **Mass:** 2369 **Score:** 9 **Expect:** 8.2e+003 **Matches:** 1
Lysozyme C (Fragment) OS=Felis catus OX=9685 GN=LYZ PE=1 SV=1
PA55H_SHEEP **Mass:** 2348 **Score:** 9 **Expect:** 8.2e+003 **Matches:** 1
Pregnancy-associated glycoprotein 55h (Fragment) OS=Ovis aries OX=9940 PE=1 SV=1

---

3. K1C10_HUMAN **Mass:** 59020 **Score:** 70 **Expect:** 0.0061 **Matches:** 18
Keratin, type I cytoskeletal 10 OS=Homo sapiens OX=9606 GN=KRT10 PE=1 SV=6
K1C10_CANLF **Mass:** 57847 **Score:** 22 **Expect:** 4.5e+002 **Matches:** 11
Keratin, type I cytoskeletal 10 OS=Canis lupus familiaris OX=9615 GN=KRT10 PE=2 SV=1
THIO_MOUSE **Mass:** 12010 **Score:** 10 **Expect:** 7.1e+003 **Matches:** 2
Thioredoxin OS=Mus musculus OX=10090 GN=Txn PE=1 SV=3
THIO_RAT **Mass:** 12008 **Score:** 10 **Expect:** 7.1e+003 **Matches:** 2
Thioredoxin OS=Rattus norvegicus OX=10116 GN=Txn PE=1 SV=2

---

4. K2C1_HUMAN **Mass:** 66170 **Score:** 69 **Expect:** 0.0089 **Matches:** 18
Keratin, type II cytoskeletal 1 OS=Homo sapiens OX=9606 GN=KRT1 PE=1 SV=6
K2C1_PANTR **Mass:** 65621 **Score:** 62 **Expect:** 0.04 **Matches:** 16
Keratin, type II cytoskeletal 1 OS=Pan troglodytes OX=9598 GN=KRT1 PE=2 SV=1
RL36_RAT **Mass:** 12317 **Score:** 10 **Expect:** 7.1e+003 **Matches:** 2
60S ribosomal protein L36 OS=Rattus norvegicus OX=10116 GN=Rpl36 PE=1 SV=2
RL36_HUMAN **Mass:** 12303 **Score:** 10 **Expect:** 7.1e+003 **Matches:** 2
60S ribosomal protein L36 OS=Homo sapiens OX=9606 GN=RPL36 PE=1 SV=3
RL36_PONAB **Mass:** 12303 **Score:** 10 **Expect:** 7.1e+003 **Matches:** 2
60S ribosomal protein L36 OS=Pongo abelii OX=9601 GN=RPL36 PE=3 SV=3
RL36_BOVIN **Mass:** 12273 **Score:** 10 **Expect:** 7.1e+003 **Matches:** 2
60S ribosomal protein L36 OS=Bos taurus OX=9913 GN=RPL36 PE=3 SV=3
COLI_CAMDR **Mass:** 3436 **Score:** 9 **Expect:** 7.6e+003 **Matches:** 1
Beta-endorphin OS=Camelus dromedarius OX=9838 GN=POMC PE=1 SV=1
PRR33_MOUSE **Mass:** 28673 **Score:** 9 **Expect:** 8.3e+003 **Matches:** 3

Proline-rich protein 33 OS=Mus musculus OX=10090 GN=Prr33 PE=2 SV=1

---

5. K1C9_HUMAN **Mass:** 62255 **Score:** 36 **Expect:** 15 **Matches:** 11
Keratin, type I cytoskeletal 9 OS=Homo sapiens OX=9606 GN=KRT9 PE=1 SV=3
PRCD_MOUSE **Mass:** 5984 **Score:** 10 **Expect:** 6.9e+003 **Matches:** 1
Photoreceptor disk component PRCD OS=Mus musculus OX=10090 GN=Prcd PE=1 SV=1

Search Parameters

Type of search : Peptide Mass Fingerprint
Enzyme : Trypsin/P
Fixed modifications : Carbamidomethyl (C)
Variable modifications : Oxidation (M)
Mass values : Monoisotopic
Protein Mass : Unrestricted
Peptide Mass Tolerance : ± 5 ppm
Peptide Charge State : 1+
Max Missed Cleavages : 2
Number of queries : 117
Selected for scoring : 84
