# Supplementary material for: Crosstalk between ORMDL3, serine palmitoyltransferase, and 5-lipoxygenase in the sphingolipid and eicosanoid metabolic pathways
Source: J Lipid Res. 2021 Sep 22;62:100121. doi: 10.1016/j.jlr.2021.100121 (PMC8527048; doi:10.1016/j.jlr.2021.100121)
Supplement: Supplemental data 2 [file mmc3.docx]

**SUPPLEMENTAL INFORMATION:**

**Crosstalk between ORMDL3, serine palmitoyltransferase, and 5-lipoxygenase in the sphingolipid and eicosanoid metabolic pathways**

Viktor Bugajev^1*^, Tomas Paulenda^1^, Pavol Utekal^1^, Michal Mrkacek^1^, Ivana Halova^1^, Ladislav Kuchar^2^, Ondrej Kuda^3^, Petra Vavrova^1^, Björn Schuster^4,5^, Sergio Fuentes-Liso^1^, Lucie Potuckova^1^, Daniel Smrz^6^, Sara Cernohouzova^1^, Lubica Draberova^1^, Monika Bambouskova^1^, and Petr Draber^1*^

^1^Department of Signal Transduction, Institute of Molecular Genetics of the Czech Academy of Sciences, Prague, Czech Republic

^2^Research Unit for Rare Diseases; Department of Pediatrics and Adolescent Medicine, First Faculty of Medicine, Charles University and General University Hospital in Prague, Prague, Czech Republic

^3^Department of Metabolism of Bioactive Lipids, Institute of Physiology of the Czech Academy of Sciences, Prague, Czech Republic

^4^Czech Centre for Phenogenomics, Institute of Molecular Genetics of the Czech Academy of Sciences, Prague, Czech Republic

^5^CZ-OPENSCREEN, Institute of Molecular Genetics of the Czech Academy of Sciences, Prague, Czech Republic

^6^Department of Immunology, Second Faculty of Medicine, Charles University, Prague, Czech Republic


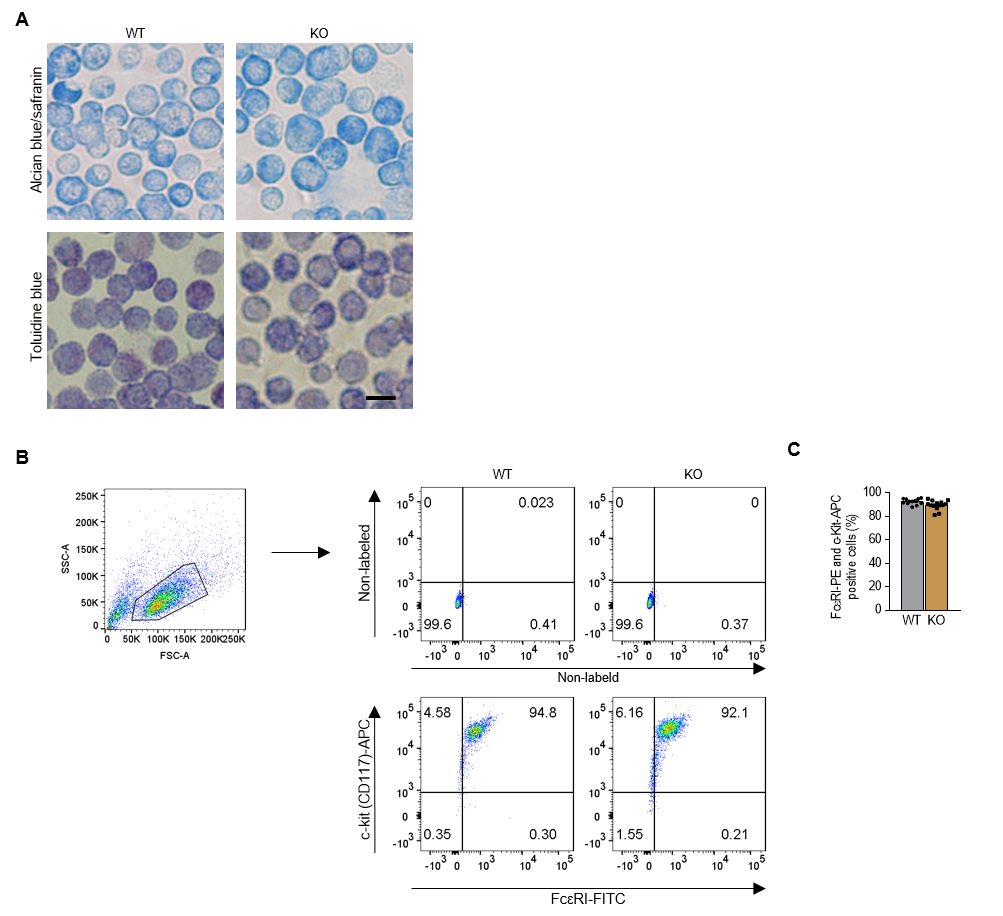


**Supplemental fig. S1. Histochemical staining and expression of FcεRI and c-Kit receptors in WT and ORMDL3 KO PDMCs.** (**A**) Alcian blue/Safranin and Toluidine blue staining of cytospin preparations of PDMCs from WT and ORMDL3 KO mice. Bar indicates 10 µm. (**B**) Flow cytometry analysis of FcεRI (FITC channel) and c-Kit (CD117; APC channel). Gating strategy is shown in the left. (**C**) Quantification of double positive WT (n = 12) and ORMDL3 KO (n = 14) cells. Quantitative data presented in C are mean ± s.e.m., calculated from n, which show numbers of biological replicates of independently isolated PDMCs. P values were determined by unpaired two-tailed Student’s *t*-test.

**
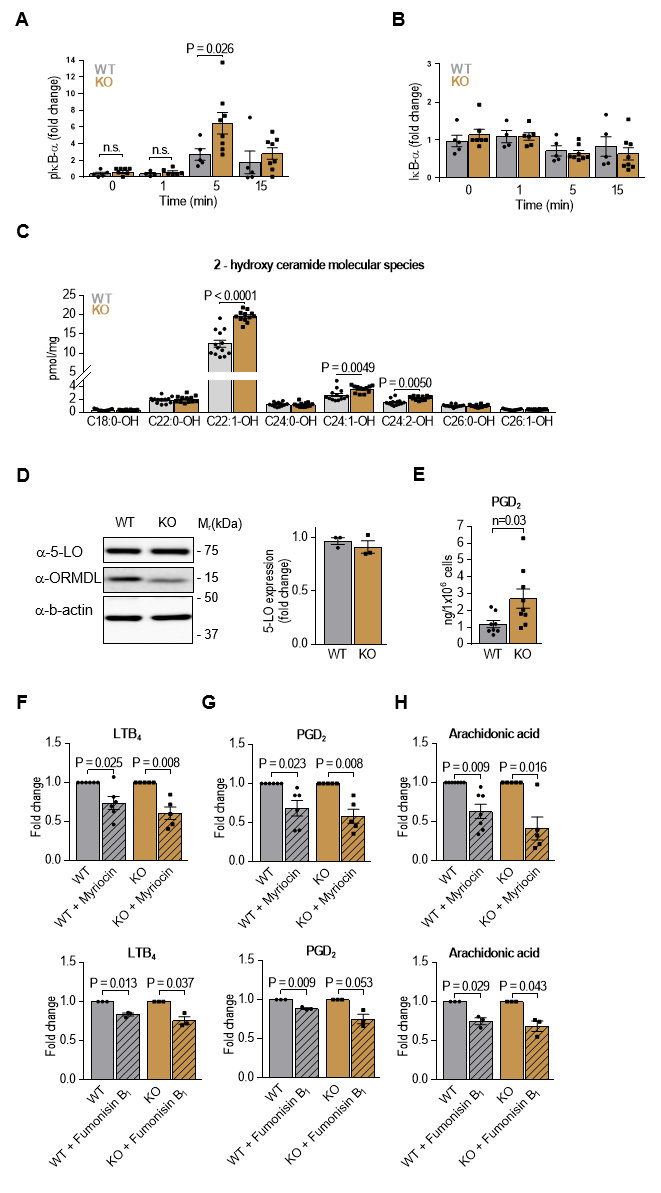
**

**Supplemental fig. S2. Statistical analysis of IκB-α basal levels and its phosphorylation, 2-hydroxy ceramide molecular species, 5-LO expression, long-lasting antigen activation-dependent PGD_2_ production, and the effect of myriocin and fumonisin B_1_ on eicosanoids in WT and ORMDL3 KO PDMCs.** (**A**) Statistical evaluation of pIκB-α levels as in **Fig. 2A** normalized to non-activated WT (n = 5) and ORMDL3 KO (n = 8) PDMCs and corresponding IκB-α load. (**B**) Statistical evaluation of IκB-α levels as in **Fig. 2A** normalized to non-activated WT (n = 5) and ORMDL3 KO (n = 8) PDMCs and corresponding β-actin load. (**C**) LC-ESI-MS/MS analysis of 2-hydroxy ceramide molecular species in resting WT (n = 13) and ORMDL3 KO (n = 12) PDMCs. (**D**) SDS-PAGE of lysates from resting WT or ORMDL3 KO PDMCs. 5-LO, ORMDL and β-actin were developed with the indicated protein-specific antibodies. The corresponding statistical analysis is shown in the right; WT (n = 3), ORMDL3 KO (n = 3) PDMCs. (**E**) UPLC MS/MS analysis of PGD_2_ released into supernatants of antigen-activated (1 ug/ml; 5 hours) WT (n = 8) and ORMDL3 KO (n = 9) PDMCs. (**F – H**) UPLC MS/MS analysis of eicosanoids in antigen-activated PDMCs non-treated or pretreated (+) with 10 μM myriocin [upper part; WT (n = 6), O3 KO (n = 5)] or 1 μM fumonisin B_1_ [bottom part; WT (n = 3), O3 KO (n = 3)]. (**F**) LTB_4_ levels. (**G**) PGD_2_ levels. (**H**) Arachidonic acid levels. Quantitative data are mean ± s.e.m., calculated from n, which show numbers of biological replicates of independently isolated PDMCs. P values were determined in A - H by unpaired two-tailed Student’s *t*-test.

**
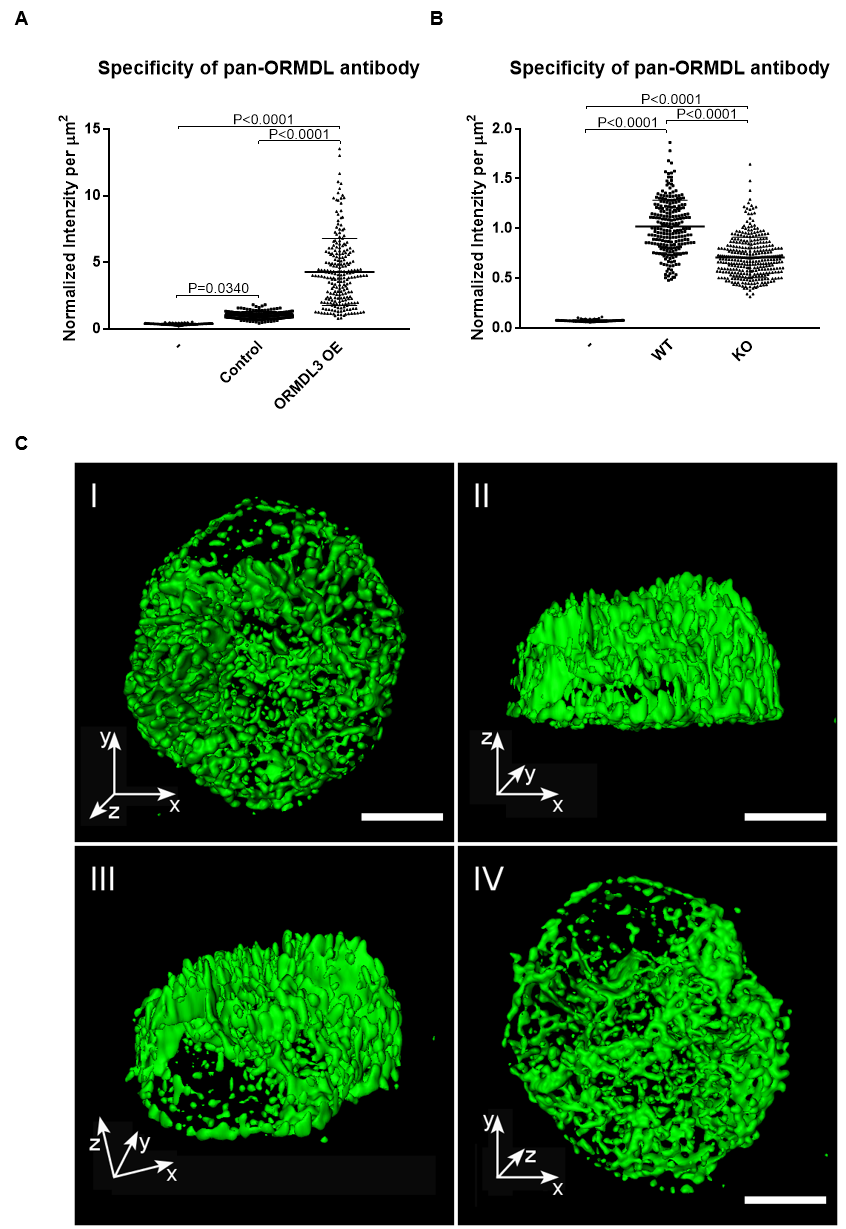
**

**Supplemental fig. S3. Specificity of pan-ORMDL antibody and the ORMDL cellular organization. (A)** Increased binding of pan-ORMDL antibody to HEK293 cells transduced with ORMDL3 when compared with cells transduced with empty vector (Control) or cells labeled only with secondary antibodies (-). **(B)** Decreased binding of pan-ORMDL antibody in PDMC with ORMDL3 KO cells when compared with WT cells or cells labeled only with secondary antibodies (-). The remaining reactivity is associated with the levels of ORMDL1 and ORMDL2. (**C**) HMC-1.1 were labeled with anti-ORMDL antibody and analyzed with confocal microscopy. Labeling with anti-ORMDL antibody resembles the tubular structure of endoplasmic reticulum, as indicated in our previous study (29). Various xyz projections are shown (I – IV). 3D surface rendering of ORMDL network. Threshold signal: 30. Resampling: 1. Scale bars represent 5µm.

**
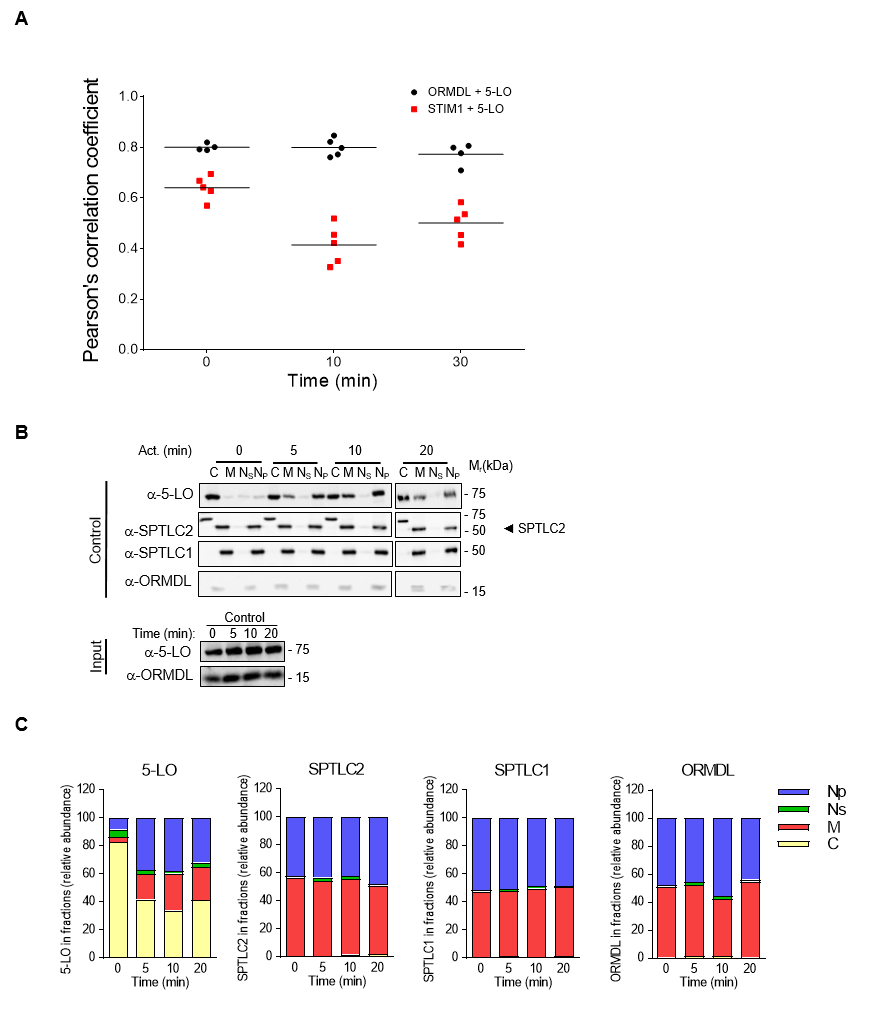
**

**Supplemental fig. S4. Extent of co-localization of 5-LO with ORMDL3 or STIM1 in HMC-1.1 cells and association of 5-LO with ER membranes in BMMCL.** (**A**) The Pearson’s correlation coefficient between endogenously expressed 5-LO and ORMDLs and between 5-LO and STIM1 at various time intervals after ionomycin activation of HMC-1. Pearson’s correlation coefficient was calculated using JACoP plugin for ImageJ. (**B**) Fractionation of resting and thapsigargin-activated BMMCL cells with ORMDL3-MYC or empty vector (Control). Cells were disrupted by nitrogen cavitation and separated into the following subcellular fractions: cytosolic (C), membrane (M), nuclear soluble (Ns), and nuclear pellet (Np). Distribution of endogenous 5-LO, SPTLC1, SPTLC2, and ORMDL family members was examined by immunoblotting. Presence of 5-LO and ORMDL in the whole lysate was also determined (Input). (**C**) Distribution of proteins in cellular compartments shown in immunoblots depicted in B. Data are representative of three independent experiments.

**
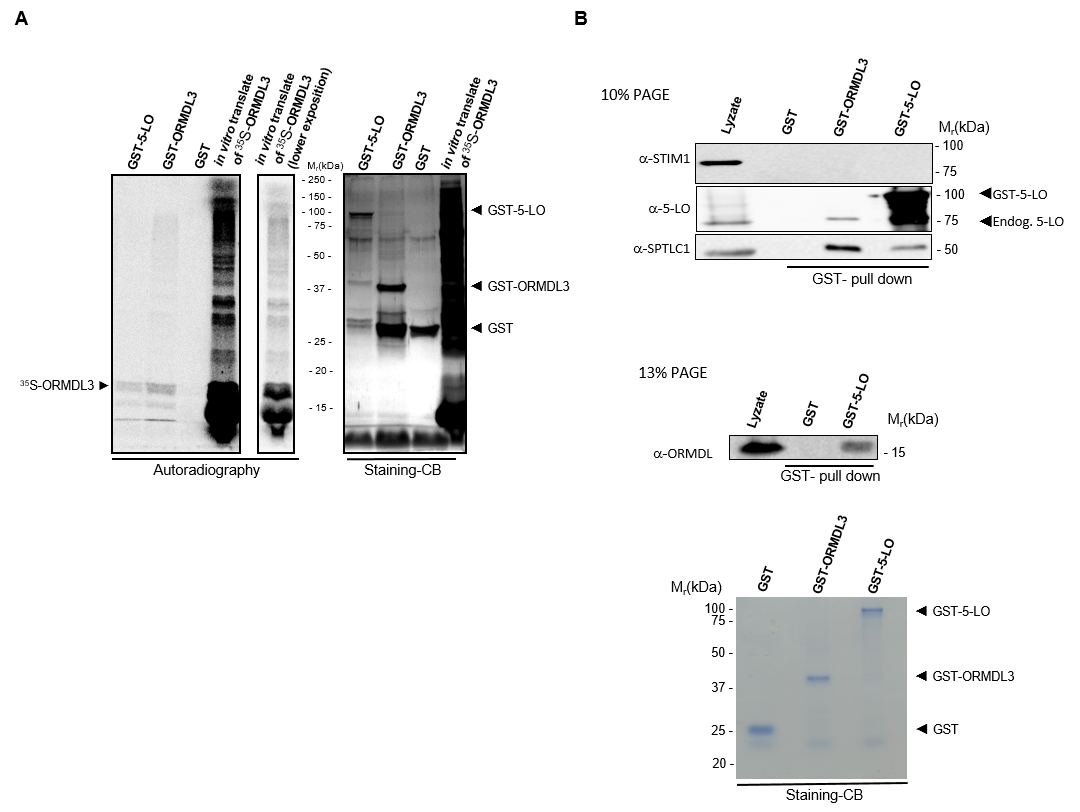
**

**Supplemental fig. S5. Association of 5-LO with ORMDL3/SPTLC1/SPTLC2 complex and ER membranes.** (**A**) Direct interaction of 5-LO with ORMDL3 as determined by GST pull-down combined with *in vitro* translation assay. ^35^S-ORMDL3 was prepared by *in vitro* translation assay and its binding to GST-5-LO, GST-ORMDL3 and GST alone was examined. SDS-PAGE followed by autoradiography was used to determine the position of radioactively labeled ^35^S-ORMDL3. The strip in the middle represents lower exposure of the ^35^S-ORMDL3 part of the gel. The same gel was stained with Coomasie Brilliant blue (CB) to determine the loading of GST-5-LO, GST-ORMDL3, and GST. (**B**) Endogenous STIM1 is not interacting with GST-5-LO or GST-ORMDL3. GST pull-downs were combined with BMMCLs lysate. Endogenous STIM1, 5-LO, SPTLC1 (10% PAGE), and ORMDL proteins (13% PAGE) were examined by immunoblotting. Coomasie Brilliant blue stained gel in the bottom shows purity and loading levels of GST, GST-ORMDL3, and GST-5-LO used in the pull down assay.


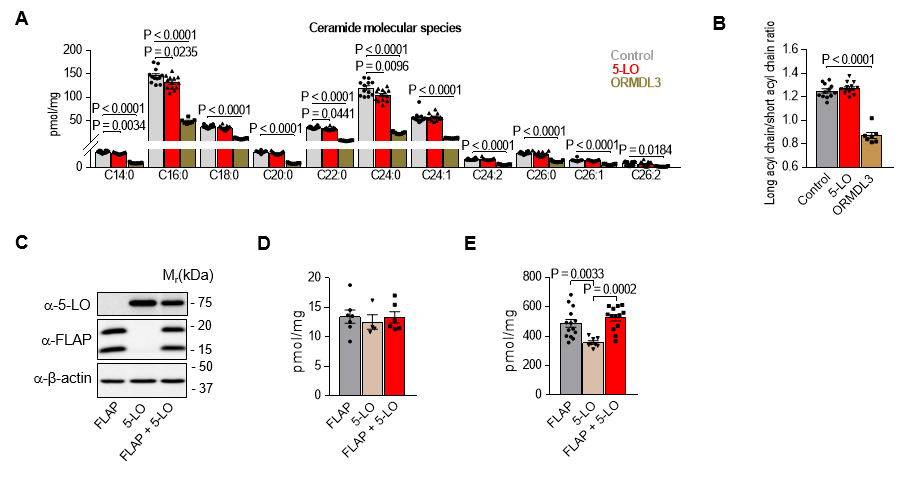


**Supplemental fig. S6. Effect of 5-LO and ORMDL3 on** **ceramide molecular species and proportions of individual ceramide molecular species in HEK293FT cells.** (**A**) LC-ESI-MS/MS analysis of non-2-hydroxy ceramide molecular species in resting HEK293FT cells stably transduced with empty vector (Control; n = 12), 5-LO (n = 13), or ORMDL3 (n = 7). (**B**) Ratio of very long acyl chains (C22:C26) and long acyl chains (C14:C20) of all molecular species calculated from data as in A. (**C**) SDS-PAGE of lysates from HEK293 cells stably transduced with murine FLAP, murine 5-LO or their combination was developed with the indicated protein-specific antibodies. (**D, E**) LC-ESI-MS/MS analysis of sphingolipids in resting HEK293 cells stably transduced with murine FLAP, murine 5-LO or their combination. (**D**) Total sphingosines, the sum of C18:1 and C18:0 is calculated; FLAP (n = 6), 5-LO (n = 4), FLAP and 5-LO (n = 6). (**E**) The sum of total ceramide molecular species (including 2-hydroxy ceramides), derived from d18:1 sphingosine, was calculated; FLAP (n = 14), 5-LO (n = 8), FLAP and 5-LO (n = 13). Quantitative data are mean ± s.e.m., calculated from n, which show numbers of biological replicates of independently transduced cells. P values were determined by one-way ANOVA with Bonferroni post hoc test.

**
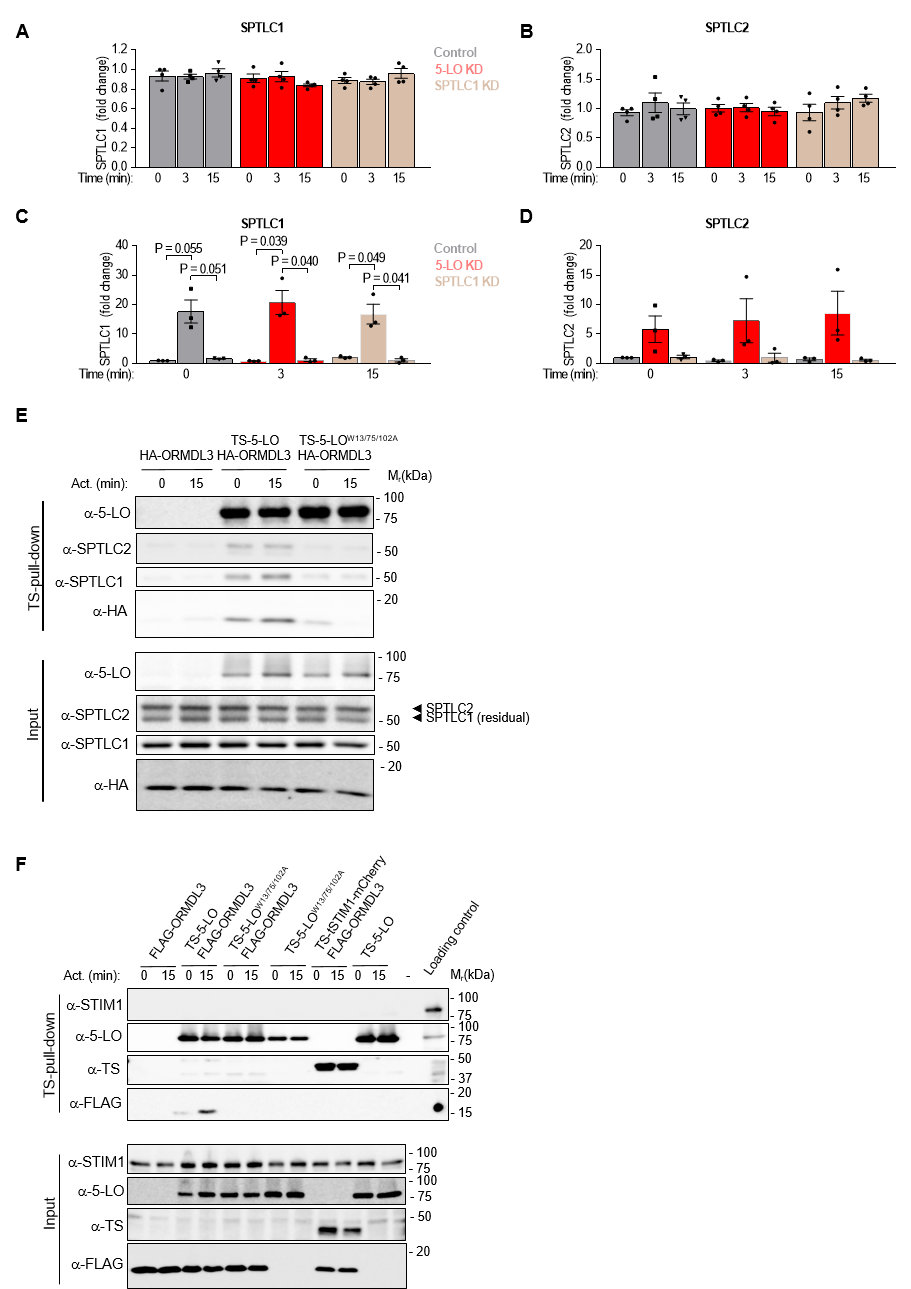
**

**Supplemental fig. S7. Impaired binding of TS-5-LO^W13/W75/W102A^ with the ORMDL3/SPTLC1/SPTLC2 complex, and endogenous STIM1 as a binding non-control.** (**A, B**) Statistical evaluation of SPTLC1 (A) and SPTLC2 (B) levels as in **Fig. 5** normalized to non-activated control BMMCL and corresponding β-actin or GRB2 load; control (n = 4), TS-5-LO (n = 4) and TS-5-LO^W13/W75/W102A^ (n = 4). (**C,** **D**) Statistical evaluation of SPTLC1 (C) and SPTLC2 (D) in pull down from BMMCL control (n = 3), TS-5-LO (n = 3) and TS-5-LO^W13/W75/W102A^ (n = 3) lysates normalized to non-activated control BMMCL and corresponding SPTLC1 (C) or SPTLC2 (D) load. (**E**) Affinity purification from post-nuclear lysates of stably transduced HEK293FT cells with HA-ORMDL3 alone or together with TS-5-LO or TS-5-LO^W13/W75/W102A^. Cells were activated for 15 min or not with thapsigargin (1 μM). Affinity-purified material (pull-down) and material load (input) were analyzed by immunoblotting with the indicated antibodies (α-). Data are representative of three independent experiments. (**F**) Affinity purification from post-nuclear lysates of stably transduced HEK293FT cells with FLAG-ORMDL3 alone or together with TS-5-LO, TS-5-LO^W13/W75/W102A^ or TS-tagged N-terminal part of STIM1-mCherry (TS-tSTIM1). TS-5-LO and TS-5-LO^W13/W75/W102A^ alone were also used. Cells were activated for 15 min or not with thapsigargin. The last line correspond to the non-activated loading control from TS-5-LO + FLAG-ORMDL3 lysate to assess the position of endogenously expressed STIM1. Affinity-purified material (pull-down) and material load (input) were analyzed by immunoblotting. Data are representative of two independent experiments. Quantitative data are mean ± s.e.m., calculated from n, which show numbers of biological replicates. P values were determined in A - D by unpaired two-tailed Student’s *t*-test.

**
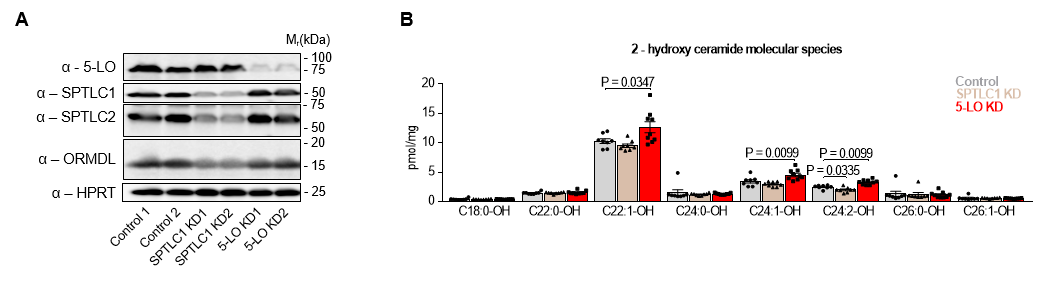
**

**Supplemental fig. S8. SPTLC1 and 5-LO knockdown (KD) in BMMCL and production of 2-hydroxy ceramide molecular species.** (**A**) Analysis of two independent SPTLC KDs (KD1 and KD2) and 5-LO KDs (KD1 and KD2) using corresponding shRNAs. Controls involved pLKO.1 vector (Control 1) and pLKO.1 with non-target shRNA (Control 2). Lysates from BMMCL were analyzed by immunoblotting with the indicated antibodies (α-). (**B**) LC-ESI-MS/MS analysis of 2-hydroxy ceramide molecular species in BMMCL resting cells transduced with Control vector (n = 10); SPTLC1 shRNA (n = 7), and 5-LO shRNA (n = 10). Quantitative data presented in B are mean ± s.e.m., calculated from n, which show numbers of biological replicates of independently transduced cells. P values were determined by one-way ANOVA with Bonferroni post hoc test.

SUPPLEMENTAL MATERIALS AND METHODS

*Plasmids.* The following cDNAs cloned in vectors used for construction of plasmids were obtained from OpenBiosystems as bacterial glycerol stocks: M. musculus cDNA ORMDL3 (ORMDL3, accession numbers: BC046594, MMM1013-9201258); M. musculus cDNA ALOX5 (5-LO; BC141213; EMM1002-99864144), H. sapiens ALOX5 (5-LO; BC132677; MHS4426-99240154); H. sapiens cDNA LTC4S (BC029498; MHS-1010-7296152), and M. musculus ALOX5AP cDNA (FLAP; BC02620; MMM1013-7510132). To express GST-tagged proteins in bacteria, cDNAs were first amplified using Phusion High-Fidelity DNA Polymerase (Thermo Fisher Scientific) and specific primers with introduced restriction sites *EcoR*I and *Not*I for ORMDL3, LTC4S, and FLAP or EcoRI and SalI for human 5-LO, allowing insertion of amplified fragments into the multiple cloning site (MCS) in pGEX-4T-2 expression vector downstream of the GST sequence. The sequence of the primers are shown in Supplementary Table 1; restriction sites are underlined. Three adenine nucleotides (nt) preceding restriction sites were added to facilitate cleavage by restriction enzymes. Two cytosines in forward primers following the restriction sites were added to preserve the reading frame. In reverse primers, STOP codons were included. For knockdown generation using lentiviral transduction, pLKO.1-puro (Control 1; Sigma-Aldrich) was used as a control in all experiments. Vector pLKO.1 containing non-target (NT) shRNA (Control 2; Sigma-Aldrich) was used as a second control when we evaluated the silencing efficiency of distinct shRNAs. A set of murine shRNAs in pLKO.1 aimed to 5-LO and SPTLC1 silencing were purchased from (Sigma-Aldrich). The most efficient silencing was achieved with vectors TRCN0000217752 and TRCN0000254912 for 5-LO and TRCN0000103400 and TRCN0000103401 for SPTLC1. For experiments focusing on production of leukotrienes and sphingolipids we used cells transduced with vectors TRCN0000217752 (5-LO shRNA) and TRCN0000103400 (SPTLC1 shRNA). For lentiviral-based protein overexpression we used pCDH-CMV-MCS-EF1-Puro (System Biosciences) or pCDH-CMV-MCS-EF1-Geneticin vector (donated by Dr. M. Koc; Institute of Molecular Genetics of the Czech Academy of Sciences, Prague, Czech Republic) as a control. Preparation of ORMDL3-MYC in pCDH-CMV-MCS-EF1-Puro was described previously (1). The ORMDL3-Twin-Strep-tag (TS) construct in pCDH-CMV-MCS-EF1-Puro was prepared by replacing the C-terminal MYC tag in ORMDL3-MYC with a TS tag. The TS tag flanked with *BamH*I and *Not*I restriction sites was generated by PCR using two opposite synthesized single-stranded DNA partially overlapping complementary at 3’ end corresponding to the middle part of the sequence encoding the TS tag. The reverse single-stranded DNA included a STOP codon. The sequence of the synthesized single-stranded DNA is shown in Supplemental Table 1 (sequence of the TS tag in bold, restriction sites underlined). The synthesized single DNA strands were heated at 95 °C for 1 min, then annealed at 65 °C for 15 s, followed by polymerization at 72 °C for 10 s using high-fidelity DNA polymerase derived from Thermococcus waitapuensis (Twa). The elongated product was loaded into wells of 3% agarose gel. The resulting band at the position of 76 bps was excised, isolated, and used for restriction enzyme cleavage and cloning into the vector. Preparation of the TS-5-LO construct in pCDH-CMV-MCS-EF1-Puro (SystemBio) was done sequentially. First, murine 5-LO was cloned into MCS using the introduced *EcoR*I or *Not*I restriction sites (see Supplemental Table 1, restriction sites underlined). Two guanines were added into forward primers to preserve the reading frame. Second, the TS tag flanked with *Xba*I and *EcoR*I restriction sites was generated by elongation of two opposite synthesized single-stranded DNAs partially overlapping complementary at 3’ end corresponding to the middle part of the sequence encoding TS tag. Elongation conditions to generate the N-terminal TS tag were identical to the conditions described for the C-terminal TS tag. The reverse single-stranded DNA comprised the Kozak consensus sequence followed by an ATG initiation codon. The sequences of synthesized single-stranded DNAs are in Supplemental Table 1 (restriction sites are underlined, TS tag sequence in bold). To clone FLAG-ORMDL3 and HA-ORMDL3 into pCDH-CMV-MCS-EF1-Puro vector (SystemBio), we used previously prepared MYC-ORMDL3 (1) by exchanging MYC tag with FLAG or HA tag using *Xba*I and *EcoR*I restriction sites. Complementary primers comprise flanking restriction sites (underlined), inserted *Xho*I restriction site, the Kozak consensus sequence followed by ATG initiation codon, and the sequence of FLAG or HA tag (see Supplemental Table 1). The corresponding reverse and forward primers were annealed by gradual temperature reduction from 95 °C to 50 °C (1 °C/min) followed by cutting using corresponding restriction enzymes, and insertion into vector upstream of ORMDL3 sequence. FLAG-ORMDL3 or HA-ORMDL3 were then excised using *Xba*I and *Not*I restriction enzymes and cloned into pCDH-CMV-MCS-EF1-Geneticin vector. The sequences of FLAG-ORMDL3 and HA-ORMDL3 were verified by sequencing.

ORMDL3, murine 5-LO, and point mutated 5-LO (see below) were also cloned into pCDH-CMV-MCS-EF1-Geneticin vector, solely without tags to examine the sphingolipid synthesis. The empty vector was used as a control. Reverse primers were the same as we used for preparation of GST-ORMDL3 and TS-5-LO constructs. Forward primers with introduced Kozak sequence are shown in Supplemental Table 1. Sequences recognized by restriction enzymes used for cloning of the insert into vector are underlined. To insert three Trp mutations into the murine 5-LO sequence at positions 13, 75, and 102, as was described elsewhere (2), we performed five consecutive PCR reactions (PCR1-5) using high-fidelity DNA polymerase derived from *Thermococcus waitapuensis* (*Twa*). All mutated Trp were encoded with a TGG nt triplet, which was changed to a GCC nt triplet encoding Ala, resulting in 5-LO^W13/75/102A^. In PCR 1, we used forward primer Trp_13_ and reverse primer Trp_75_. Forward primer Trp_13_ introduces mutation W13A because it bears the GCC mutation (for Ala) at this position; reverse primer Trp_13_ carries the W75A mutation at position Trp_75_. PCR 2 used forward primer Trp_75_, which includes mutation at position 75 and is partially complementary to the Trp_75_ reverse primer and Trp_102_ reverse primer (including mutation at position 102). PCR 3 used forward primer Trp_102_ and reverse primer (R_3_) placed to the end of 5-LO cDNA and contained no mutation. Since products of all three PCRs are partially complementary, they were used simultaneously as templates for PCR No.4. Forward primer Trp_13_ and reverse primer R_3_ were used. The resulting product encompasses the whole sequence of murine 5-LO cDNA bearing all three mutations (5-LO ^W13/75/102A^). The product of PCR4 was amplified in PCR5 with primers used for cloning of murine 5-LO into the pCDH vector. The resulting product was introduced into TS-5-LO-pCDH-CMV-MCS-EF1-Puro plasmid after excision of WT murine 5-LO using restriction enzymes *EcoR*I and *Not*I to leave the sequence encoding TS tag in the vector. In this way, we obtained construct TS-5-LO^W13/75/102A^ suitable for lentiviral transduction. Primers used for PCR1-3 with underlined sequences of insertion encoding the Ala GCC triplet instead of WT Trp TGG triplet are shown in Supplemental Table 1. The insertion of truncated version of STIM1 into pCDH vector containing SA-tag at the C-terminus, we used primers containing *Xba*I or *BamH*I restriction enzymes targeting the N-terminus of STIM1 labeled with mCherry (behind signal peptide) including transmembrane domain. All primers were ordered from Sigma-Aldrich. All restriction enzymes were ordered from New England BioLabs.

Reference List

1. Bugajev, V., Halova, I., Draberova, L., Bambouskova, M., Potuckova, L., Draberova, H., Paulenda, T., Junyent, S., and Draber, P. 2016. Negative regulatory roles of ORMDL3 in the FceRI-triggered expression of proinflammatory mediators and chemotactic response in murine mast cells. *Cell Mol. Life Sci.* **73:** 1265-1285.

2. Kulkarni, S., Das, S., Funk, C. D., Murray, D., and Cho, W. 2002. Molecular basis of the specific subcellular localization of the C2-like domain of 5-lipoxygenase. *J. Biol. Chem.* **277:** 13167-13174.
