# Supplementary material for: Crosstalk between ORMDL3, serine palmitoyltransferase, and 5-lipoxygenase in the sphingolipid and eicosanoid metabolic pathways
Source: J Lipid Res. 2021 Sep 22;62:100121. doi: 10.1016/j.jlr.2021.100121 (PMC8527048; doi:10.1016/j.jlr.2021.100121)
Supplement: Supplemental data 4 [file mmc5.pdf]

MATRIX SCIENCE MASCOT Search Results

Protein View: LOX5_MOUSE

Polyunsaturated fatty acid 5-lipoxygenase OS=Mus musculus OX=10090 GN=Alox5 PE=1 SV=3

**Database:**	Uniprot
**Score:**	81
**Expect:**	0.00049
**Monoisotopic mass (M_r):**	78602
**Calculated pI:**	5.78
**Taxonomy:**	Mus musculus

Sequence similarity is available as an NCBI BLAST search of LOX5_MOUSE against nr.

Search parameters

**MS data file:**	D23.mgf
**Enzyme:**	Trypsin/P: cuts C-term side of KR.
**Fixed modifications:**	Carbamidomethyl (C)
**Variable modifications:**	Oxidation (M)
**Mass values searched:**	117
**Mass values matched:**	21

Protein sequence coverage: 35%

Matched peptides shown in ***bold red***.

1 MPSYTVTVAT GSQWFAGTDD YIYLSLIGSA GCSEKHLLDK **AFYNDFERGA**

51 **VDSYDVTVDE** **ELGEIYLVK**I EKR**KYWLHDD** **WYLK**YITLK**T** **PHGDYIEFPC**

101 **YRWITGEGEI** **VLR**DGRAKLA RDDQIHILKQ HRRKELEARQ KQYRWMEWNP

151 GFPLSIDAKC HKDLPRDIQF DSEK**GVDFVL** **NYSKAMENLF** **INRFMHMFQS**

201 **SWHDFADFEK** IFVKISNTIS ERVKNHWQED LMFGYQFLNG CNPVLIKRCT

251 ALPPK**LPVTT** **EMVECSLER**Q LSLEQEVQEG NIFIVDYELL DGIDANKTDP

301 CTHQFLAAPI CLLYKNLANK IVPIAIQLNQ TPGESNPIFL PTDSKYDWLL

351 AKIWVRSSDF HVHQTITHLL R**THLVSEVFG** **IAMYRQLPAV** **HPLFK**LLVAH

401 VRFTIAINTK AR**EQLICEYG** **LFDKANATGG** **GGHVQMVQRA** **VQDLTYSSLC**

451 **FPEAIK**AR**GM** **DSTEDIPFYF** **YR**DDGLLVWE AIQSFTMEVV SIYYENDQVV

501 EEDQELQDFV K**DVYVYGMR**G KKASGFPKSI KSREKLSEYL TVVIFTASAQ

551 HAAVNFGQYD WCSWIPNAPP TMRAPPPTAK **GVVTIEQIVD** **TLPDR**GRSCW

601 HLGAVWALSQ FQENELFLGM YPEEHFIEKP VKEAMIRFRK **NLEAIVSVIA**

651 ERNKNKKLPY YYLSPDRIPN SVAI

Unformatted sequence string: 674 residues (for pasting into other applications).

Sort by ◉ residue number ○ increasing mass ○ decreasing mass
Show ◉ matched peptides only ○ predicted peptides also

Start – End	Observed	Mr(expt)	Mr(calc)	ppm	M	Peptide
41 – 48	1061.4694	1060.4621	1060.4614	0.71	0	K.AFYNDFER.G
49 – 69	2314.1257	2313.1185	2313.1264	-3.42	0	R.GAVDSYDVTVDEELGEIYLVK.I
74 – 84	1566.7790	1565.7717	1565.7667	3.22	1	R.KYWLHDDWYLK.Y
75 – 84	1438.6768	1437.6695	1437.6717	-1.53	0	K.YWLHDDWYLK.Y
90 – 102	1654.7303	1653.7231	1653.7246	-0.92	0	K.TPHGDYIEFPCYR.W
103 – 113	1272.6941	1271.6868	1271.6874	-0.45	0	R.WITGEGEIVLR.D
175 – 184	1141.5905	1140.5832	1140.5815	1.50	0	K.GVDFVLNYSK.A
185 – 193	1123.5581	1122.5508	1122.5491	1.48	0	K.AMENLFINR.F + Oxidation (M)
194 – 210	2221.9049	2220.8976	2220.9034	-2.58	0	R.FMHMFQSSWHDFADFEK.I + 2 Oxidation (M)
256 – 269	1679.7946	1678.7873	1678.7906	-1.97	0	K.LPVTTEMVECSLER.Q + Oxidation (M)
372 – 385	1622.8331	1621.8258	1621.8286	-1.76	0	R.THLVSEVFGIAMYR.Q
386 – 395	1149.6787	1148.6714	1148.6706	0.70	0	R.QLPAVHPLFK.L
413 – 424	1514.7120	1513.7047	1513.7123	-4.99	0	R.EQLICEYGLFDK.A
425 – 439	1498.7159	1497.7086	1497.7107	-1.40	0	K.ANATGGGGHVQMVQR.A + Oxidation (M)
440 – 456	1941.9575	1940.9502	1940.9554	-2.64	0	R.AVQDLTYSSLCFPEAIK.A
459 – 472	1756.7489	1755.7417	1755.7450	-1.93	0	R.GMDSTEDIPFYFYR.D + Oxidation (M)
512 – 519	1018.4686	1017.4613	1017.4590	2.30	0	K.DVYVYGMR.G + Oxidation (M)
581 – 595	1654.8998	1653.8925	1653.8938	-0.77	0	K.GVVTIEQIVDTLPDR.G
641 – 652	1313.7431	1312.7359	1312.7350	0.64	0	K.NLEAIVSVIAER.N
657 – 667	1414.7376	1413.7303	1413.7292	0.76	1	K.KLPYYYLSPDR.I
658 – 667	1286.6417	1285.6344	1285.6343	0.12	0	K.LPYYYLSPDR.I

No match to: 842.5103, 973.5280, 998.5301, 1044.0991, 1051.7162, 1082.0530, 1090.5309, 1107.5395, 1109.4912, 1118.5094, 1122.6311, 1143.6637, 1157.5931, 1165.5831, 1165.7492, 1179.6003, 1193.6160, 1234.6804, 1235.5278, 1249.1099, 1265.6389, 1271.0884, 1277.7102, 1300.5308, 1302.7148, 1307.6794, 1320.5820, 1323.6714, 1327.7578, 1330.7629, 1340.6619, 1348.5823, 1356.6611, 1357.7238, 1365.6378, 1381.6426, 1383.6893, 1393.7321, 1423.6841, 1434.7688, 1475.7454, 1475.7925, 1493.7344, 1505.6810, 1584.7099, 1603.8225, 1638.8662, 1664.7910, 1707.7706, 1716.8505, 1729.9444, 1741.7578, 1762.8620, 1765.7324, 1791.7260, 1797.0119, 1807.9162, 1811.9146, 1837.9732, 1838.9041, 1851.9212, 1859.9639, 1887.9709, 1934.0120, 1964.0663, 1968.8552, 1972.0113, 1981.9973, 1993.9737, 1996.9743, 2083.0339, 2096.0424, 2117.0253, 2150.0785, 2211.1074, 2225.1171, 2239.1399, 2256.0461, 2257.9590, 2283.1813, 2297.2067, 2342.9882, 2367.2645, 2383.9569, 2399.0148, 2510.1341, 2541.3305, 2565.1688, 2692.4308, 2705.1590, 2717.0912, 2808.3220, 2872.3841, 2902.3992, 3223.2832, 3312.3144

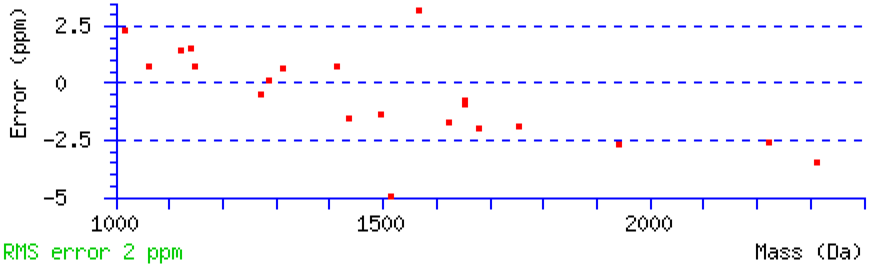

RMS error 2 ppm

Mascot: http://www.matrixscience.com/
